# Melatonin Mitigates Sarcopenic Obesity via Microbiota and Short‐Chain Fatty Acids: Evidence From Epidemiologic and In Vivo Studies

**DOI:** 10.1002/jcsm.13869

**Published:** 2025-06-13

**Authors:** Xiaoxing Mo, Lihui Shen, Xinyu Wang, Wenqing Ni, Linyan Li, Lili Xia, Hongjie Liu, Ruijie Cheng, Lin Wen, Jian Xu, Liegang Liu

**Affiliations:** ^1^ Department of Nutrition and Food Hygiene, Hubei Key Laboratory of Food Nutrition and Safety, MOE Key Lab of Environment and Health, School of Public Health, Tongji Medical College Huazhong University of Science and Technology Wuhan China; ^2^ Department of Elderly Health Management Shenzhen Center for Chronic Disease Control Shenzhen Guangdong China

**Keywords:** gut microbiota, melatonin, oxidative stress, protein synthesis, sarcopenic obesity, short‐chain fatty acids

## Abstract

**Introduction:**

Gut dysbiosis is closely related to the development of sarcopenic obesity (SO). Melatonin (MLT) regulates gut microbiota and promotes the production of short‐chain fatty acids (SCFAs). However, whether MLT affects SO through the gut microbiota and SCFAs remains unclear. This study aimed to investigate the effect and mechanism of MLT in SO induced by a high‐fat diet (HFD).

**Methods:**

First, a case‐control study was conducted to explore the potential association between serum MLT levels and SO‐related parameters in 31 patients. Next, rats fed with a HFD were orally administered with MLT for 16 weeks, and obesity‐related metabolic disorders and muscle atrophy were measured. 16S rRNA gene sequencing and gas chromatography–mass spectrometry were used to detect gut microbiota and SCFAs, respectively. Gut barrier integrity was assessed by the expression of the Muc‐2 protein and tight junction proteins. Finally, faecal microbiota transplantation and SCFAs administration were performed to confirm the causal role of the gut microbiota and SCFAs in the effect of MLT on SO.

**Results:**

The serum levels of MLT decreased in patients with SO (29.87 ± 6.71 vs. 24.94 ± 5.68, *p* < 0.01) and were closely associated with appendicular skeletal muscle mass index (*r* = 0.3514, *p* < 0.01) and handgrip strength (*r* = 0.2824, *p* < 0.05). MLT ameliorated obesity‐related metabolic disorders (*p* < 0.05), poor muscle mass (*p* < 0.05), strength (*p* < 0.05) and function (*p* < 0.05) and muscle atrophy (*p* < 0.05) in HFD‐fed rats. MLT regulated HFD‐induced gut dysbiosis, which was mainly characterized by increases in SCFAs‐related bacteria and SCFAs (*p* < 0.05). MLT recovered HFD‐induced impairment of gut barrier integrity by promoting the expression levels of Muc‐2, claudin‐1, occludin and zonula occluden‐1 proteins in the colon (*p* < 0.05). Correlation analysis showed that SCFAs‐related bacteria and SCFAs were negatively associated with SO. Faecal suspension from MLT‐treated rats promoted the production of SCFAs in recipient rats (*p* < 0.05). In addition, faecal suspension from MLT‐treated rats partially mitigated metabolic disorders (*p* < 0.05), poor muscle mass and function (*p* < 0.05) and muscle atrophy (*p* < 0.05) in recipient rats. SCFAs treatment alleviated the development of SO in HFD‐fed rats by suppressing metabolic disorders (*p* < 0.05), reducing muscle oxidative stress and inflammation (*p* < 0.05) and promoting protein synthesis through the AKT/mTOR/p70S6k signalling pathway (*p* < 0.05).

**Conclusions:**

MLT mitigated HFD‐induced SO by regulating the gut microbiota and promoting the production of SCFAs. MLT might be a novel strategy for delaying the progression of SO.

## 1 Introduction

Sarcopenia, which is characterized by poor muscle mass, strength and function, has become a major public issue that endangers the physical health of older adults. With the increasing consumption of high‐fat diet (HFD) in older adults, sarcopenic obesity (SO), defined as the combination of obesity and sarcopenia, has attracted worldwide attention because of its high risk of disabilities, fractures, metabolic diseases or even death [1]. Although multiple efforts have been made to prevent sarcopenia or obesity, evidence on the preventive intervention strategy for SO remains insufficient [2]. Thus, novel effective strategies should be developed to prevent and treat SO.

The gut microbiota and its metabolites are implicated in the development of SO; in particular, their dysbiosis has been discovered in SO‐related animal models and humans [3, 4]. Emerging evidence has suggested that HFD‐induced dysbiosis of the gut microbiota and its metabolites triggers muscle inflammation and oxidative stress, thereby reducing muscle protein synthesis and inducing muscle atrophy [5, 6]. Supplementation with probiotics or prebiotics improves muscle mass, strength and function and reduces visceral fat mass in db/db mice by regulating gut dysbiosis and its related proinflammatory responses [7, 8]. Therefore, strategies targeting gut dysbiosis provide novel treatment options for SO.

Melatonin (MLT), which can be isolated from the pineal gland, possesses multiple physiological properties, such as anti‐insomnia, antioxidant, and anti‐inflammatory [9]. Epidemiological studies have indicated that MLT improves muscle strength and physical performance in older adults [10, 11]. In addition, MLT and exercise for 8 weeks alleviate poor muscle mass and function in HFD‐fed SAMP8 mice [12]. Although the relationship between MLT and sarcopenia has been reported, the effect of MLT on SO has been rarely discussed. Recent evidence suggests that MLT affects various diseases by modulating the gut microbiota [13, 14]. MLT also promotes the production of short‐chain fatty acids (SCFAs) [15]. SCFAs are typical microbial metabolites that regulate inflammatory responses and oxidative stress and may exhibit antagonistic effects on obesity or muscle atrophy [16, 17]. However, whether MLT affects SO through the gut microbiota and SCFAs remains unclear.

This study first explored the potential association between MLT and SO through a case‐control study, and then determined the effect and mechanism of MLT on SO from the perspectives of the gut microbiota and SCFAs in vivo. Faecal microbiota transplantation (FMT) and SCFAs treatment were conducted to explore the crucial role of the gut microbiota and SCFAs in mediating the effect of MLT on SO. The signalling pathway through which SCFAs regulated by MLT affects SO was also explored.

## 2 Methods

### 2.1 Case‐Control Study

Older adults with SO were recruited from the baseline population of the Shenzhen Ageing Cohort Study (SZ‐ageing). The details of this cohort study were reported in our previous work [S1]. In short, 3000 participants were initially recruited and underwent comprehensive assessments including general physical examinations, blood tests, dual‐energy x‐ray absorptiometry (DXA) scans and face‐to‐face questionnaire surveys. After excluding 418 participants with missing data (including DXA scans, anthropometric measurements, handgrip strength and physical performance data) and insufficient serum samples, we further excluded 1380 participants based on the following criteria: (1) individuals who took sleeping pills, probiotics or prebiotics within 4 weeks; (2) individuals who had trauma injury, spinal injury or any disease that may affect physical function, such as kidney disease, malignant tumours, severe liver disease or thyroid cancer, etc.; (3) individuals who required hormone therapy; (4) individuals who had infection diseases, tumours or mental and autoimmune diseases, such as cognitive impairment, Alzheimer's disease, multiple sclerosis, myasthenia gravis, etc.; and (5) individuals who were unable to cooperate due to other factors. Ultimately, we included 31 older adults with SO as cases and 31 age‐ and sex‐matched controls without SO for analysis. Blood samples were collected from the participants at 8:00 am–8:30 am after overnight fasting. The samples were centrifuged at 3000 rpm for 15 min to obtain serum.

### 2.2 Definitions of SO

SO was defined as the coexistence of sarcopenia and obesity. The diagnosis of sarcopenia was based on the Asian Working Group for Sarcopenia (AWGS) 2019, and obesity is defined using body fat percentage (BF%) (≥ 25% for men, ≥ 35% for women) [S2].

According to the AWGS 2019, sarcopenia was defined as low skeletal muscle mass and low muscle strength or/and low physical performance with the following criteria: (1) appendicular skeletal muscle mass index (ASMI, appendicular skeletal muscle mass/height squared) < 7.0 kg/m^2^ for men and < 5.7 kg/m^2^ for women is defined as low skeletal muscle mass, as assessed by a DXA scan; (2) handgrip strength < 26 kg for men and < 18 kg for women is defined as low muscle strength, as assessed by a hydraulic dynamometer model (Jamar, Preston, Jackson, Missouri, USA); and (3) short physical performance battery (SPPB) score ≤ 9 is defined as low physical performance, and the test includes three components: gait speed test (0–4 score), chair stand test (0–4 score), and four‐stage balance test (0–4 score).

This case‐control study has been registered in the Chinese Clinical Trial Registry (ChiCTR2200060055) and has obtained approval from the ethics committee of the Shenzhen Center for Chronic Disease Control (SZCCC‐ 2022‐001‐01‐ PJ).

### 2.3 Animals

Specific pathogen‐free (SPF) 12‐month‐old female Sprague–Dawley (SD) rats were obtained from the Vital River Laboratory Animal Center (Beijing, China) and housed in a SPF facility (22°C ± 2°C, 50% ± 5% and 12 h dark–12 h light cycle). Female rats were selected for two reasons: (1) epidemiological evidence indicated that females had higher risk of developing SO and poorer prognosis than males [S3], and (2) our prior research demonstrated that long‐term HFD feeding led to SO in female rats [6]. The rats were given free access to food and water and acclimated in the environment for 1 week before the experiment. Animal experiments were approved by the Institutional Animal Care and Use Committee at Tongji Medical College, Huazhong University of Science and Technology and performed in accordance with the National Research Council's Guide for the Care and Use of Laboratory Animals (Permission ID: 4534).

### 2.4 MLT Preparation

MLT (HY‐ B0075, Med Chem Express) was dissolved in pure ethanol (1%, v/v) and diluted in normal saline at the final concentration of 10 mg/kg. The dosage of MLT intervention was chosen based on existing evidence of its protective effect against sarcopenia [S4] and obesity [S5, S6].

### 2.5 MLT Intervention

Twenty‐four 12‐month‐old female rats were randomly allocated into the Control (*n* = 8), HFD (*n* = 8), and HFD + MLT (*n* = 8) groups. Rats in the Control group were fed with a normal diet (AIN‐93 M, 10% kcal from fat, research diet) and received 10 mg/kg normal saline containing (1%, v/v) ethanol daily through oral administration for 16 weeks. The remaining rats were fed with HFD (D12451, 45% kcal from fat, research diet) and received 10 mg/kg normal saline containing (1%, v/v) ethanol (for the HFD group) or 10 mg/kg MLT containing (1%, v/v) ethanol (for the HFD + MLT group) daily through oral administration for 16 weeks. Body weight was measured twice a week, and food intake was recorded daily. At the end of the experiment, faeces were collected, and the rats were subjected to oral glucose tolerance test (OGTT), intraperitoneal insulin tolerance test (ipITT) and behaviour tests. Three rats from each group were randomly selected for magnetic resonance imaging (MRI). After overnight fasting, the rats were anaesthetised with isoflurane (5% v/v) and euthanized by extracting blood from the descendent aorta. Blood was centrifuged at 3000 rpm for 15 min to obtain serum. The extensor digitorum longus (EDL) and soleus (SOL) muscles of four rats from each group were randomly selected and fixed with 4% paraformaldehyde for pathological examination. The remaining EDL and SOL muscles were collected for other analyses. EDL and SOL were selected because they are regarded as typical fast‐switch and slow‐switch muscles, respectively [S7].

### 2.6 Faecal Collection and Faecal Suspension Preparation

Each rat was placed in a sterile cage until defecation. Faeces were collected into sterile centrifuge tubes for 16S rRNA gene sequencing and faecal suspension preparation. The preparation of faecal suspension was conducted as described in our previous work [S8]. In brief, 100 mg of faeces was added with 1.5 mL of sterile phosphate buffered saline (PBS), vortexed for 5 min and centrifuged at 2500 rpm for 2 min. The supernatant (1 mL) was mixed with 10% glycerol and stored at −80°C before FMT. This process was carried out on an anaerobic workstation and controlled within 1 h.

### 2.7 FMT Intervention

To further illustrate the beneficial effects of MLT mediated by the gut microbiota and its effects on the host circulating SCFAs, we performed FMT experiment by transferring the microbiota from MLT‐treated rats to conventional recipient rats. FMT was implemented as described in our previous work [S8]. Twenty‐four 12‐month‐old female recipient rats were randomly allocated into the FMT control (FMTC, *n* = 8), FMT HFD (FMTH, *n* = 8), and FMT HFD + MLT (FMTHM, *n* = 8) groups and orally administered with the faecal suspension (1 mL) of rats in the control group, rats in the HFD group and rats in the HFD + MLT group, respectively. The recipient rats were fed with a normal diet (AIN‐93 M, 10% kcal from fat, research diet) and received the faecal suspension twice a week for 16 weeks. Body weight was measured twice a week and food intake was recorded daily. At the end of the experiment, faeces were collected and the rats were subjected to OGTT, ipITT and behaviour tests. Three rats from each group were randomly selected for MRI. After overnight fasting, the rats were anaesthetised with isoflurane (5% v/v) and euthanized by extracting blood from the descendent aorta. Blood was centrifuged at 3000 rpm for 15 min to obtain serum. The EDL and SOL muscles of four rats from each group were randomly selected and fixed with 4% paraformaldehyde for pathological examination. The remaining EDL and SOL muscles were collected for other analyses.

### 2.8 SCFAs Intervention

An additional animal study was conducted to investigate whether MLT‐mediated improvement in SO was associated with modulation of gut microbial metabolites, particularly SCFAs, which were reduced in HFD‐fed rats and restored by MLT intervention. Sixteen 12‐month‐old female rats were randomly allocated into the HFD (*n* = 8) and HFD + SCFAs (*n* = 8) groups. The rats were fed with HFD (D12451, 45% kcal from fat, research diet) and received normal drinking water (for HFD group) or SCFAs mixture (for HFD + SCFAs group) daily for 16 weeks. Sodium acetate, sodium butyrate, and sodium propionate were dissolved in drinking water to prepare SCFAs mixture with the final concentration of 67.5 mM acetate, 40 mM butyrate and 25.9 mM propionate. The drinking water was replaced every 2 days. Body weight was measured twice a week, and the intake of food and water was recorded daily. The dosage of SCFAs for intervention was chosen based on the existing evidence of its protective effect on muscle mass and function [S9]. At the end of the experiment, the rats were subjected to OGTT, ipITT and behaviour tests. Three rats from each group were randomly selected for MRI. After overnight fasting, the rats were anaesthetised with isoflurane (5% v/v) and euthanized by extracting blood from the descendent aorta. Blood was centrifuged at 3000 rpm for 15 min to obtain serum. The EDL and SOL muscles of four rats from each group were randomly selected and fixed with 4% paraformaldehyde for pathological examination. The EDL and SOL muscles of three rats from each group were randomly selected for transmission electron microscopy examination. The remaining SOL and EDL muscles were collected for other analyses.

### 2.9 Statistical Analysis

Data were tested for normality and homogeneity of variance before downstream analysis. Parametric data were analyzed by t‐test (for two groups) or one‐way ANOVA with Tukey's test (for three groups). Nonparametric data were analyzed by Mann–Whitney *U*‐test (for two groups) or Kruskal–Wallis test with Dunn's multiple comparison post‐test (for three groups). Correlation analysis was conducted by Spearman's correlation analysis. The data from 16S rRNA gene sequencing were analyzed by R software (version 4.3.0), and other data analyses were performed on SPSS 29.0. Heatmaps of differences in gut microbiota composition were created by the ggplot2 R package, and other figures were created by Graphpad Prism 9.4.0. *p* < 0.05 was regarded as significant. Data were reported as mean ± SEM The sample size was shown in each method and figure legend. Additional methods were available in Data S1.

## 3 Results

### 3.1 Decreased Serum MLT Levels in Patients With SO

**FIGURE 1:**
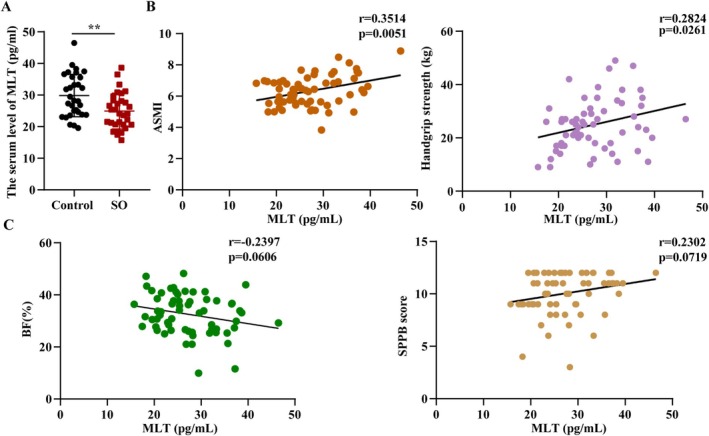
**SO patients exhibit reduction in serum MLT levels.** (A) The box plot showed serum levels of MLT in non‐SO control and SO patients (*n* = 31/group, repeated three times). (B) Correlation between MLT levels and appendicular skeletal muscle mass index (ASMI) and handgrip strength. (C) Correlation between MLT levels and body fat BF percentage (BF%) and short physical performance battery (SPPB) core. Data were reported as mean ± SEM and analyzed by T‐test (A). Correlation analysis was assessed by Spearman's correlation analysis (B, C). **p < 0.01 versus non‐SO control.

The BF% of the patients with SO increased, whereas their ASMI, handgrip strength, and SPPB score decreased compared with the control group (Table S2, all *p* < 0.05). The serum levels of MLT decreased in patients with SO compared with the control group (Figure 1A, Control vs. SO, 29.87 ± 6.71 vs. 24.94 ± 5.68, *p* < 0.01). The potential interaction between MLT and SO‐related parameters, including BF%, ASMI, handgrip strength and SPPB score, was further performed. The serum levels of MLT were positively associated with ASMI (*r* = 0.3514, *p* < 0.01; Figure 1B) and handgrip strength (*r* = 0.2824, *p* < 0.05; Figure 1B). Meanwhile, the serum levels of MLT were negatively associated with BF% and positively associated with SPPB score without statistical significance (Figure 1C, both *p* > 0.05). These findings revealed that deficiency of MLT levels was implicated in the development of SO.

### 3.2 MLT Ameliorates Obesity‐Related Metabolic Disorders in HFD‐Fed Rats

The levels of serum MLT were reduced in HFD‐fed rats compared with those in rats fed with a normal diet. However, HFD‐induced decreases in the levels of serum MLT were improved by MLT intervention (Figure S1A, *p* < 0.05). We found no significant difference in food intake among the three groups (Figure S1B, *p* > 0.05). HFD significantly increased the body weight of rats, whereas HFD‐induced body weight gain was reduced by MLT (Figure S1C, *p* < 0.05). HFD‐induced increases in the body fats of rats were also counteracted by MLT, as revealed by MRI (Figure S2A, *p* < 0.05). In addition, HFD‐induced increases in the levels of total cholesterol (TC), triglyceride (TG) and low‐density lipoprotein cholesterol (LDL‐C) in serum were reduced by MLT (Figure S2B, *p* < 0.05). In OGTT and ipITT, the level of blood glucose and the value of area under the curve (AUC) were elevated in HFD‐fed rats (Figure S2C,D, *p* < 0.05). However, the HFD‐induced increases in the level of blood glucose and the value of AUC were reduced by MLT (Figure S2C,D, *p* < 0.05). Overall, HFD‐induced obesity, dyslipidemia, impaired glucose tolerance and low insulin sensitivity were suppressed by MLT.

### 3.3 MLT Alleviates Poor Muscle Mass, Strength and Function in HFD‐Fed Rats

**FIGURE 2:**
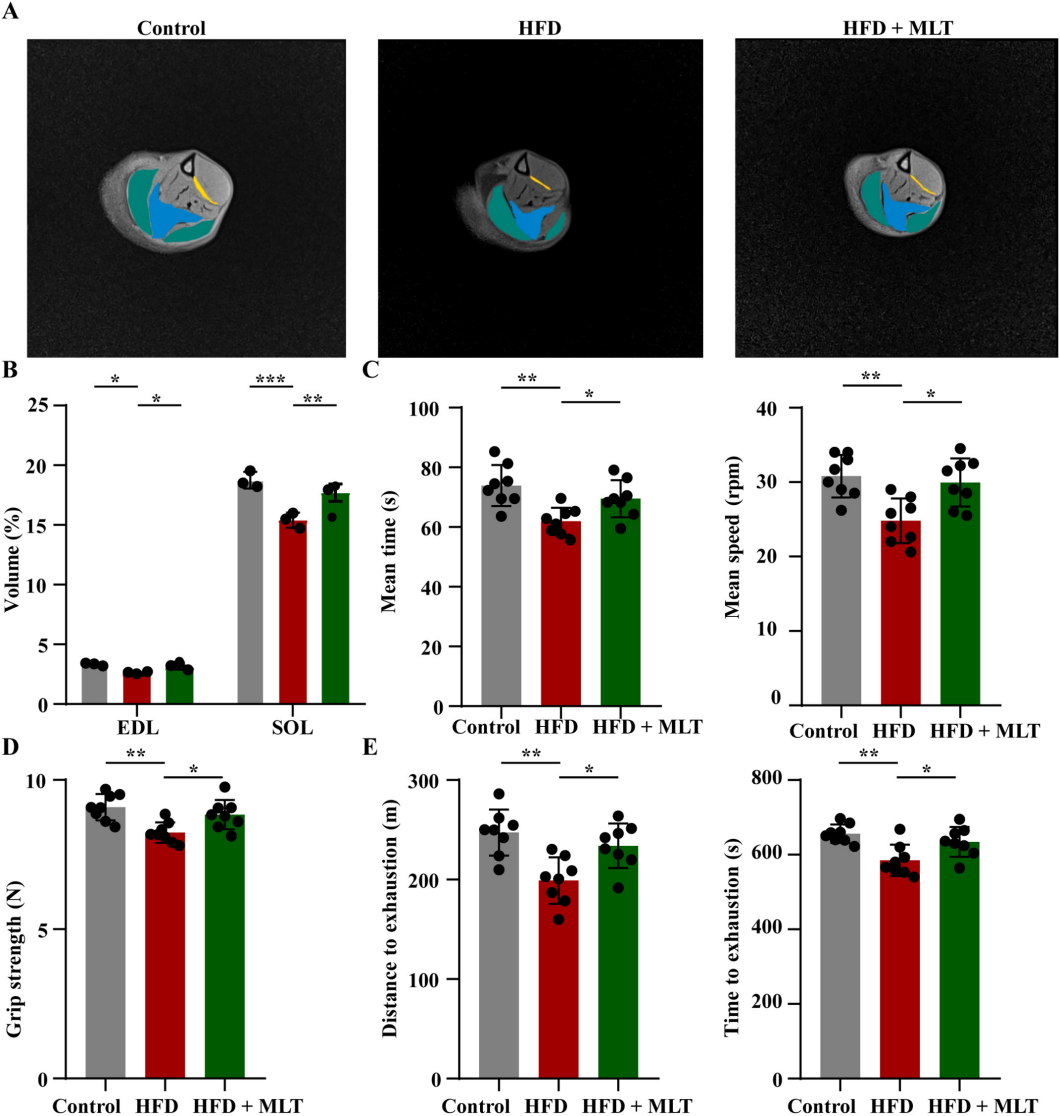
**MLT ameliorates poor muscle mass, strength, and function in HFD‐fed rats.** (A) The representative images of gastrocnemius, extensor digitorum longus (EDL) and soleus (SOL) muscles examined by magnetic resonance imaging (MRI) (*n* = 3 rats/group). Green represents gastrocnemius, blue represents SOL and yellow represents EDL. (B) Quantitative analysis of muscle volume (*n* = 3 rats/group). (C) The mean time and speed of rats in the rotarod test (*n* = 8 rats/group). (D) Grip strength (*n* = 8 rats/group). (E) The total distance and total time of rats in the exhaustive running test (*n* = 8 rats/group). Data were reported as mean ± SEMand analyzed by one‐way ANOVA, followed by Tukey's multiple comparisons test (B–E). *p < 0.05, **p < 0.01, ***p < 0.001 versus HFD‐fed rats.

As indicated by MRI examination, the volumes of EDL and SOL muscles were reduced in HFD‐fed rats compared with those in rats fed with a normal diet. However, HFD‐induced decreases in the volumes of EDL and SOL muscles were improved by MLT (Figure 2A,B, *p* < 0.05). Similarly, HFD‐induced decreases in the weights of EDL and SOL muscles were elevated by MLT (Figure S3A, *p* < 0.05). In the rotarod test, HFD‐fed rats displayed decreased average time and average speed compared with rats fed with a normal diet. Conversely, MLT alleviated the decreases in average time and average speed caused by HFD (Figure 2C, *p* < 0.05). The grip strength decreased in HFD‐fed rats compared with that in rats fed with a normal diet, whereas the HFD‐induced reduction in grip strength was enhanced by MLT (Figure 2D, *p* < 0.05). In the exhaustive running test, HFD‐fed rats showed reductions in the distance and time of exhaustion compared with rats fed with a normal diet. By contrast, MLT increased the distance and time of exhaustion in HFD‐fed rats (Figure 2E, *p* < 0.05). Overall, HFD‐induced poor muscle mass, strength and function were ameliorated by MLT.

### 3.4 MLT Reduces Muscle Atrophy in HFD‐Fed Rats

**FIGURE 3:**
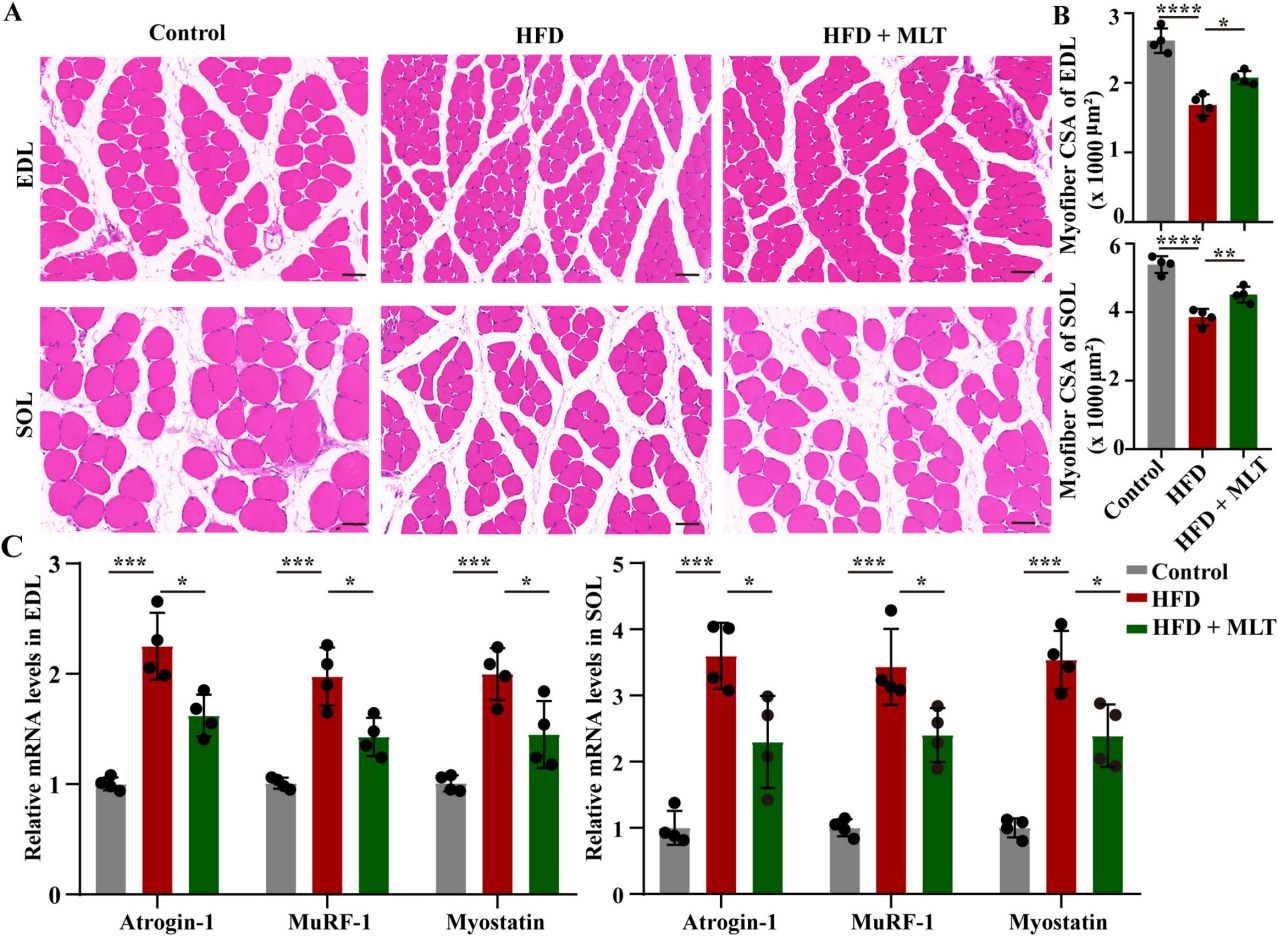
**MLT mitigates muscle atrophy in HFD‐fed rats.** (A) The representative images of Haematoxylin and eosin (H&E) staining of EDL and SOL muscles (*n* = 4 rats/group), scale bar = 50 μm. (B) The cross‐sectional area (CSA) of EDL and SOL muscles (*n* = 4 rats/group). (C) The mRNA expression levels of Atrogin‐1, MuRF‐1, and Myostatin in EDL and SOL muscles (*n* = 4 rats/group, repeated 3 times). Data were reported as mean ± SEM and analyzed by one‐way ANOVA, followed by Tukey's multiple comparisons test (B, C). *p < 0.05, **p < 0.01, ***p < 0.001, ****p < 0.0001 versus HFD‐fed rats.

The results of H&E staining revealed that the cross‐sectional area (CSA) of EDL and SOL muscles was lower in HFD‐fed rats than in rats fed with a normal diet. However, the CSA of EDL and SOL muscles was higher in MLT‐treated rats than that in HFD‐fed rats (Figure 3A,B, *p* < 0.05). Meanwhile, the mRNA levels of muscle atrophy factors, including atrogin‐1, MuRF‐1 and myostatin, were elevated in HFD‐fed rats. However, HFD‐induced increases in the mRNA levels of atrogin‐1, MuRF‐1 and myostatin were reduced by MLT (Figure 3C, *p* < 0.05). Overall, HFD‐induced muscle atrophy was inhibited by MLT.

### 3.5 MLT Mitigates the Downregulation of Muscle Protein Synthesis in HFD‐Fed Rats

The expression levels of p‐Akt^Ser473^/Akt, p‐mTOR^Ser2448^/mTOR, and p‐p70S6k^Thr389^/p70S6k in EDL and SOL muscles were downregulated in HFD‐fed rats compared with those in rats fed with a normal diet. Conversely, HFD‐induced decreases in the expression levels of p‐Akt^Ser473^/Akt, p‐mTOR^Ser2448^/mTOR, and p‐p70S6k^Thr389^/p70S6k in EDL and SOL muscles were upregulated by MLT (Figure S4A,B, all *p* < 0.01). Overall, HFD‐induced decrease in muscle protein synthesis was alleviated by MLT.

### 3.6 MLT Modulates Microbiota Composition in HFD‐Fed Rats

**FIGURE 4:**
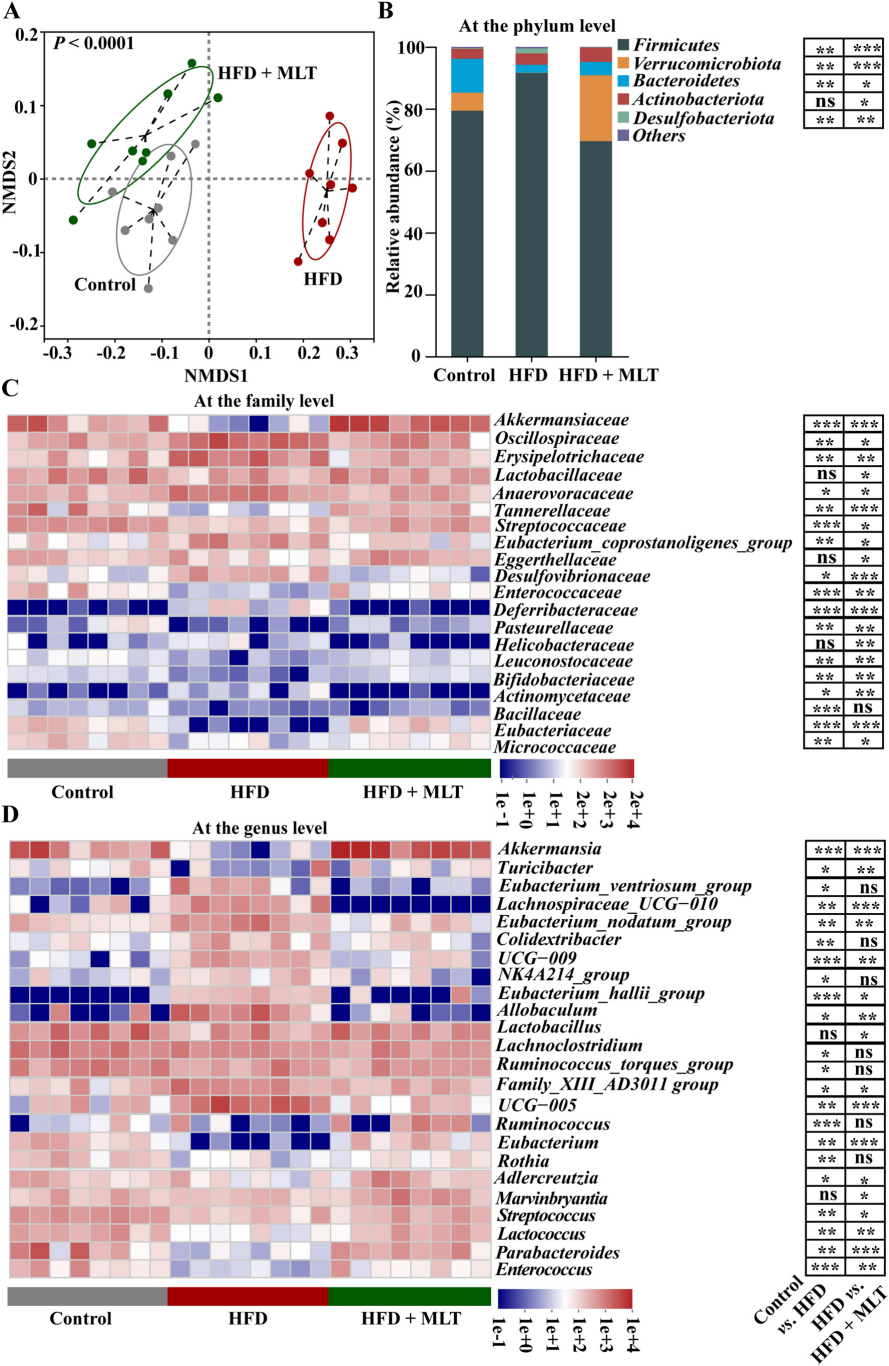
**MLT modulates gut dysbiosis in HFD‐fed rats.** (A) The β‐diversity of gut microbiota based on non‐metric multidimensional scaling (NMDS) analysis (*n* = 8 rats/group). (B) The relative abundance of gut microbiota at the phylum level (*n* = 8 rats/group). (C) Heatmap of gut microbiota at the family level (*n* = 8 rats/group). (D) Heatmap of gut microbiota at the genus level (*n* = 8 rats/group). Data were analyzed by Kruskal–Wallis test, followed by Dunn's Multiple Comparison post‐test (B–D). *P*‐values were adjusted for false discovery rate (FDR) using the Benjamini‐Hochberg method, with adjusted *p* < 0.05 considered statistically significant. *p < 0.05, **p < 0.01, ***p < 0.001 versus HFD‐fed rats. ns: not significant.

Non‐metric multidimensional scaling (NMDS) analysis revealed that the composition and structure of the gut microbiota significantly differed among the three groups (Figure 4A, *p* < 0.05). At the phylum level, the relative abundance of *Firmicutes* and *Desulfobacteriota* increased and that of *Verrucomicrobiota* and *Bacteroidetes* decreased in HFD‐fed rats compared with those in rats fed with a normal diet (Figure 4B, *p* < 0.05). However, HFD‐induced alterations in the gut microbiota were alleviated by MLT (Figure 4B, *p* < 0.05). At the family level, HFD‐induced increase in the relative abundance of bacteria, such as *Oscillospiraceae*, *Anaerovoracaceae*, *Erysipelotrichaceae* and *Eubacterium_coprostanoligenes_group* and decrease in the relative abundance of bacteria, such as *Akkermansiaceae Lactobacillaceae*, *Eubacteriaceae*, *Streptococcaceae*, *Micrococcaceae* and *Leuconostocaceae* were attenuated by MLT (Figure 4C, *p* < 0.05). Correspondingly, at the genus level, HFD‐induced increase in the relative abundance of deleterious bacteria, such as *Lachnospiraceae_UCG−010*, *Eubacterium_nodatum*_*group*, *UCG−009*, and *Eubacterium_hallii*_*group*, was reduced by MLT (Figure 4D, *p* < 0.05). Meanwhile, HFD‐induced reduction in the relative abundance of beneficial bacteria, such as *Akkermansia*, *Ruminococcus*, *Lactobacillus*, *Adlercreutzia*, *Marvinbryantia* and *Lactococcus*, was enhanced by MLT. Overall, HFD‐induced gut dysbiosis was restored by MLT.

### 3.7 MLT Promotes SCFAs Production in HFD‐Fed Rats

**FIGURE 5:**
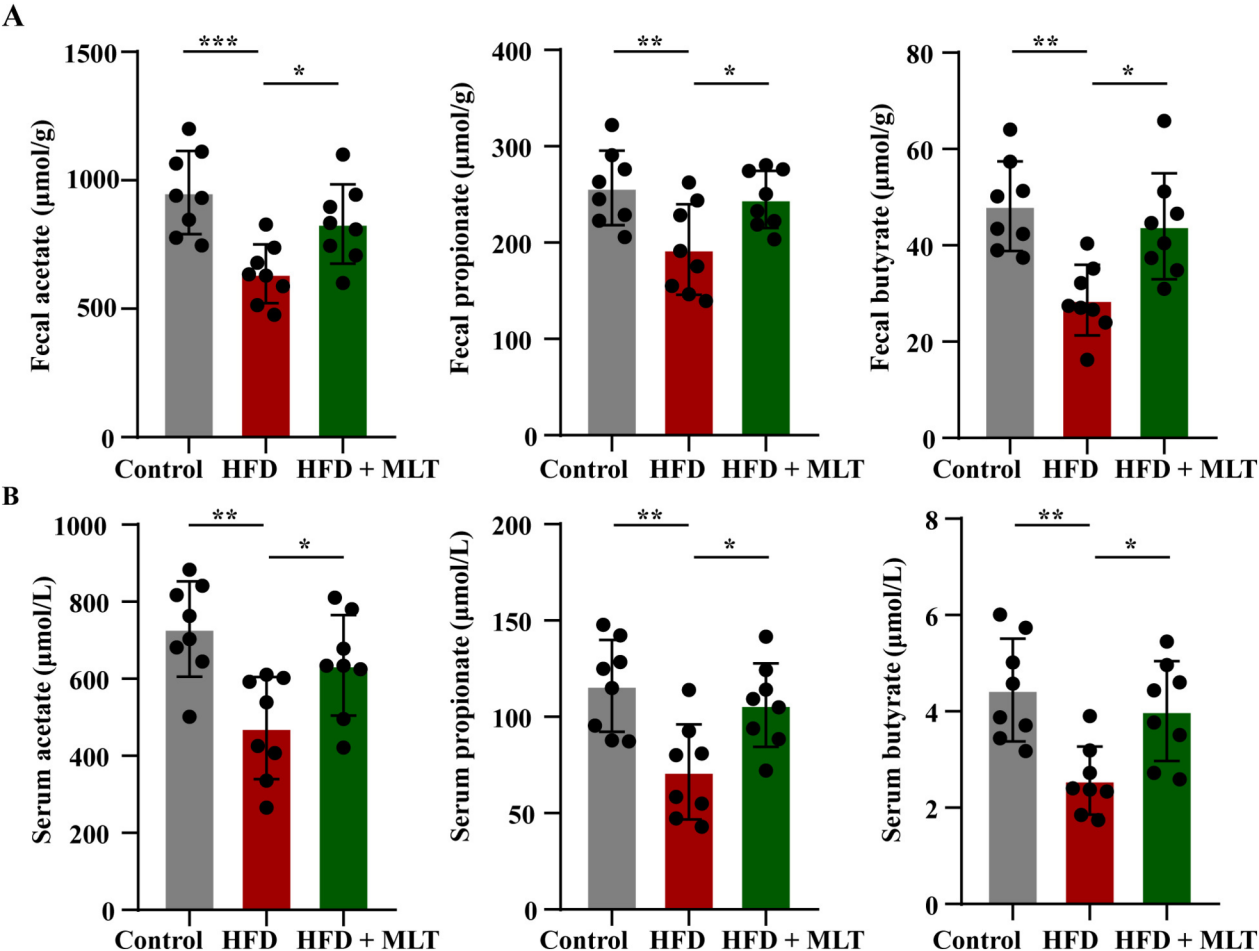
**MLT promotes the production of SCFAs in HFD‐fed rats.** (A) The levels of acetate, propionate and butyrate in faeces (*n* = 8 rats/group). (B) The levels of acetate, propionate, and butyrate in serum (*n* = 8 rats/group). Data were reported as mean ± SEM and analyzed by one‐way ANOVA, followed by Tukey's multiple comparisons test (A, B). *p < 0.05, **p < 0.01, ***p < 0.001 versus HFD‐fed rats.

HFD significantly reduced the levels of SCFAs, including acetate, propionate, and butyrate in faeces, whereas HFD‐induced reductions in the levels of acetate, propionate, and butyrate were elevated by MLT (Figure 5A, all *p* < 0.05). Similarly, HFD‐induced decreases in the levels of acetate, propionate, and butyrate in serum were mitigated by MLT (Figure 5B, all *p* < 0.05). Overall, MLT increased the levels of SCFAs in faeces and serum in HFD‐fed rats.

### 3.8 MLT Renovates gut Barrier Impairment in HFD‐Fed Rats

The mucin (Muc)‐2 protein and tight junction proteins are the main components of the gut barrier [S10]. As indicated by immunofluorescence staining, HFD significantly decreased the immunofluorescence intensity of Muc‐2 in the colon. Conversely, HFD‐induced decrease in the immunofluorescence intensity of Muc‐2 in the colon was alleviated by MLT (Figure S5A,B, all *p* < 0.05). In addition, HFD significantly decreased the expression levels of claudin‐1, occludin, and zonula occluden (Zo)‐1 proteins in the colon. However, HFD‐induced downregulation in the expression levels of claudin‐1, occludin, and Zo‐1 proteins in the colon were upregulated by MLT (Figure S5C,D, all *p* < 0.01). Overall, HFD‐induced gut barrier impairment was recovered by MLT.

### 3.9 Association Among Microbiota, SCFAs, and SO

We firstly investigated the association between gut microbiota and SCFAs. As shown in Figure S6A, a positive association was observed between *Akkermansia*, *Ruminococcus*, *Lactobacillus*, *Adlercreutzia*, *Marvinbryantia* and *Lactococcus* and SCFAs. A negative association was observed between *Lachnospiraceae_UCG−010 Eubacterium_nodatum*_*group*, *UCG−009*, and *Eubacterium_hallii_group* and SCFAs. Next, we examined the association among microbiota, SCFAs and SO. As depicted in Figure S6B, a positive association was observed between *Lachnospiraceae_UCG−010*, *Eubacterium_nodatum*_*group*, *UCG−009*, and *Eubacterium_hallii*_*group* and SO. A positive association was also observed between *Akkermansia*, *Ruminococcus*, *Lactobacillus*, *Adlercreutzia*, *Marvinbryantia*, *Lactococcus* and SCFAs and muscle mass, strength and function. Meanwhile, a negative association was observed between *Akkermansia*, *Ruminococcus*, *Lactobacillus*, *Adlercreutzia*, *Marvinbryantia*, *Lactococcus* and SCFAs and SO. A negative association was also observed between *Lachnospiraceae_UCG−010*, *Eubacterium_nodatum*_*group*, *UCG−009* and *Eubacterium_hallii*_*group* and muscle mass, strength and function. Overall, MLT‐related alterations in the gut microbiota and SCFAs were closely associated with SO.

### 3.10 Faecal Microbiota of MLT‐Treated Rats Attenuates Metabolic Disorders and Muscle Atrophy in Recipient Rats

**FIGURE 6:**
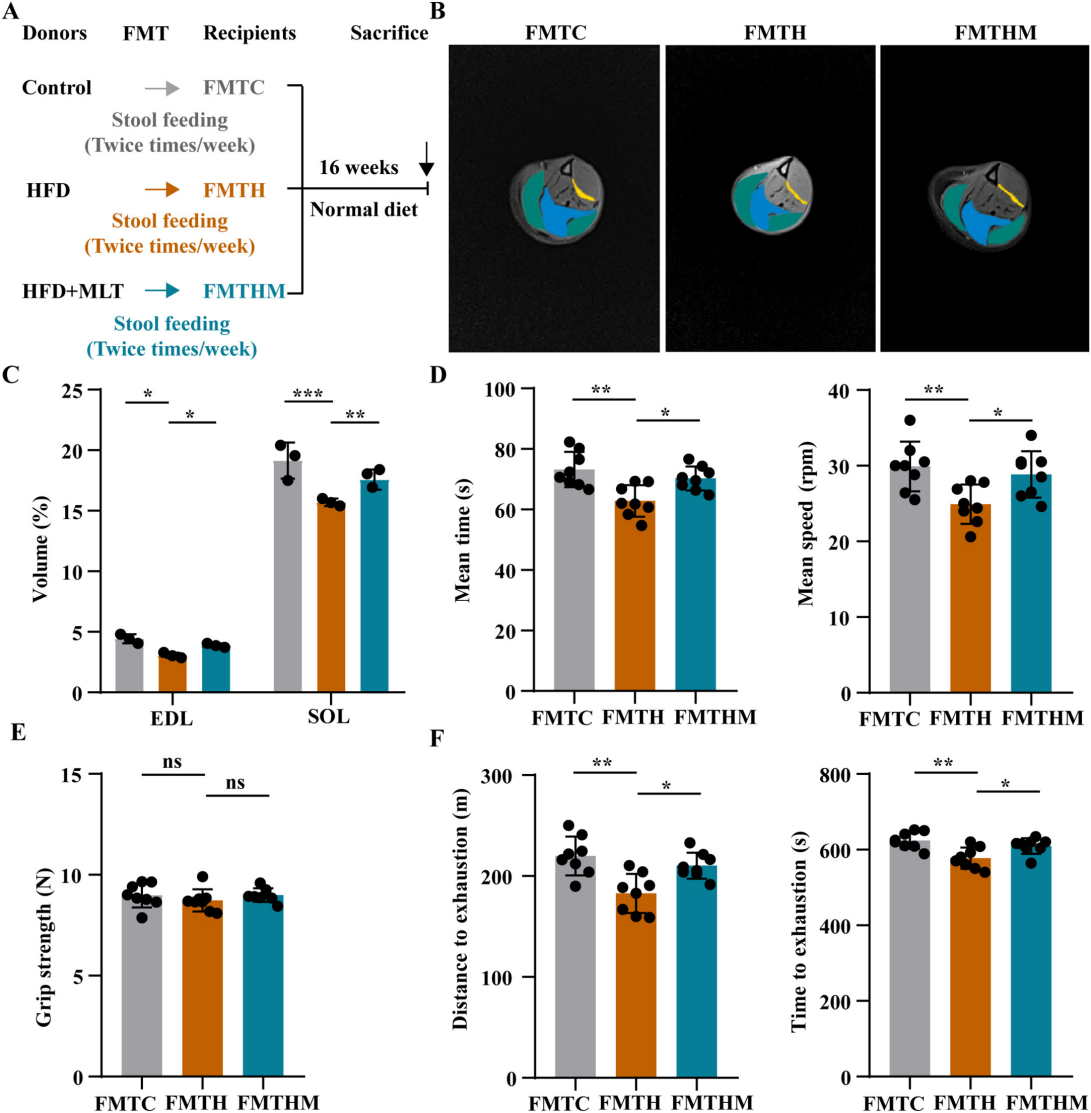
**Faecal suspension of MLT‐treated rats attenuates poor muscle mass and function in recipient rats.** (A) Study design of faecal microbiota transplantation experiment. (B) The representative images of gastrocnemius, EDL and SOL muscles examined by MRI (*n* = 3 rats/group). Green represents gastrocnemius, blue represents SOL and yellow represents EDL. (C) Quantitative analysis of muscle volume (*n* = 3 rats/group). (D) The mean time and speed of rats in the rotarod test (*n* = 8 rats/group). (E) Grip strength (*n* = 8 rats/group). (F) The total distance and total time of rats in the exhaustive running test (*n* = 8 rats/group). Data were reported as mean ± SEM and analyzed by one‐way ANOVA, followed by Tukey's multiple comparisons test (C–F). *p < 0.05, **p < 0.01, ***p < 0.001 versus the recipient rats received faecal suspension of HFD‐fed rats. ns: not significant.

FMT was used to investigate the causal role of the gut microbiota in the effect of MLT on SO. After FMT intervention, the gut microbiota of the recipient rats was similar to that of the donor rats (Figure S7A,D). The faecal microbiota of HFD‐fed rats decreased the levels of acetate, propionate, and butyrate in faeces and serum. Conversely, the faecal microbiota of MLT‐treated rats promoted the production of acetate, propionate, and butyrate in faeces and serum in recipient rats (Figure S8A,B, all *p* < 0.05). FMT had no significant effect on the body weight and food intake (Figure S9A,B). The faecal microbiota of HFD‐fed rats caused glucose and lipid metabolism disorders, poor muscle mass and function and muscle atrophy in recipient rats, whereas these changes were suppressed by the faecal microbiota of MLT‐treated rats (Figure 6 and Figures S10–S12, all *p* < 0.05). Overall, the faecal microbiota of HFD‐fed rats caused glucose and lipid metabolism disorders and muscle atrophy in recipient rats, and this effect was alleviated by the faecal microbiota of MLT‐treated rats.

### 3.11 SCFAs Mitigate SO by Reducing Metabolic Disorders, Muscle Oxidative Stress and Inflammation and Promoting Muscle Protein Synthesis

**FIGURE 7:**
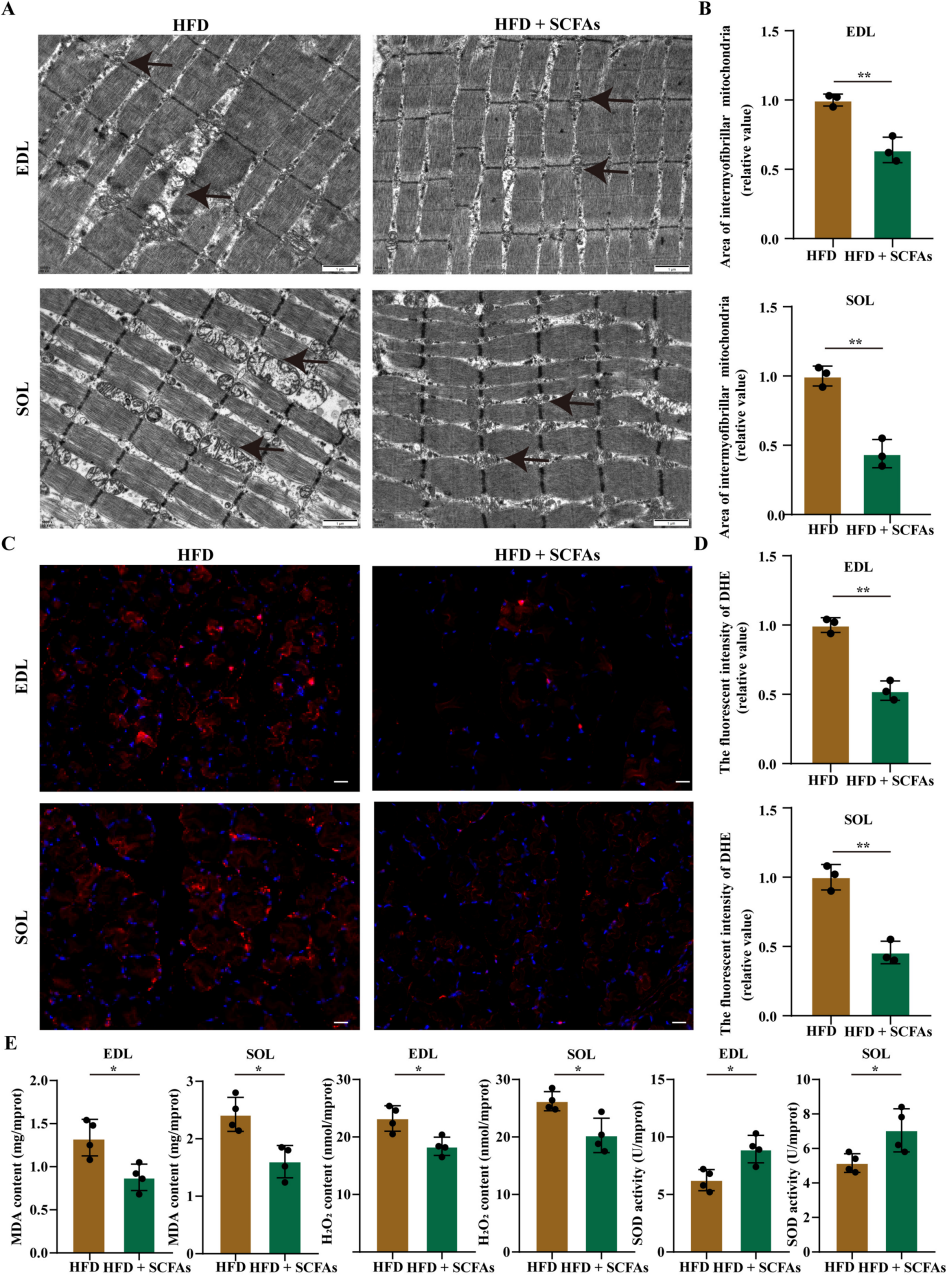
**SCFAs treatment reduces muscle ROS production and muscle oxidative stress in HFD‐fed rats.** (A) Representative images of intermyofibrillar mitochondria from transmission electron micrographs in EDL and SOL muscles (scale bar, 1 μm) (*n* = 3 rats/group). Arrows represent mitochondria. (B) Quantification of mitochondria area in EDL and SOL muscles (*n* = 3 rats/group). (C) Representative images of muscle sections with dihydroethidium (DHE) in EDL and SOL muscles (scale bar, 20 μm) (*n* = 3 rats/group). (D) The fluorescence intensity of DHE in EDL and SOL muscles (*n* = 3 rats/group). (E) The levels of oxidative stress biomarkers, including malondialdehyde (MDA), H_2_O_2_, and superoxide dismutase (SOD) in EDL and SOL muscles (*n* = 4 rats/group, repeated three times). Data were reported as mean ± SEM and analyzed by T‐test (B, D, E). *p < 0.05, **p < 0.01 versus HFD‐fed rats.

**FIGURE 8:**
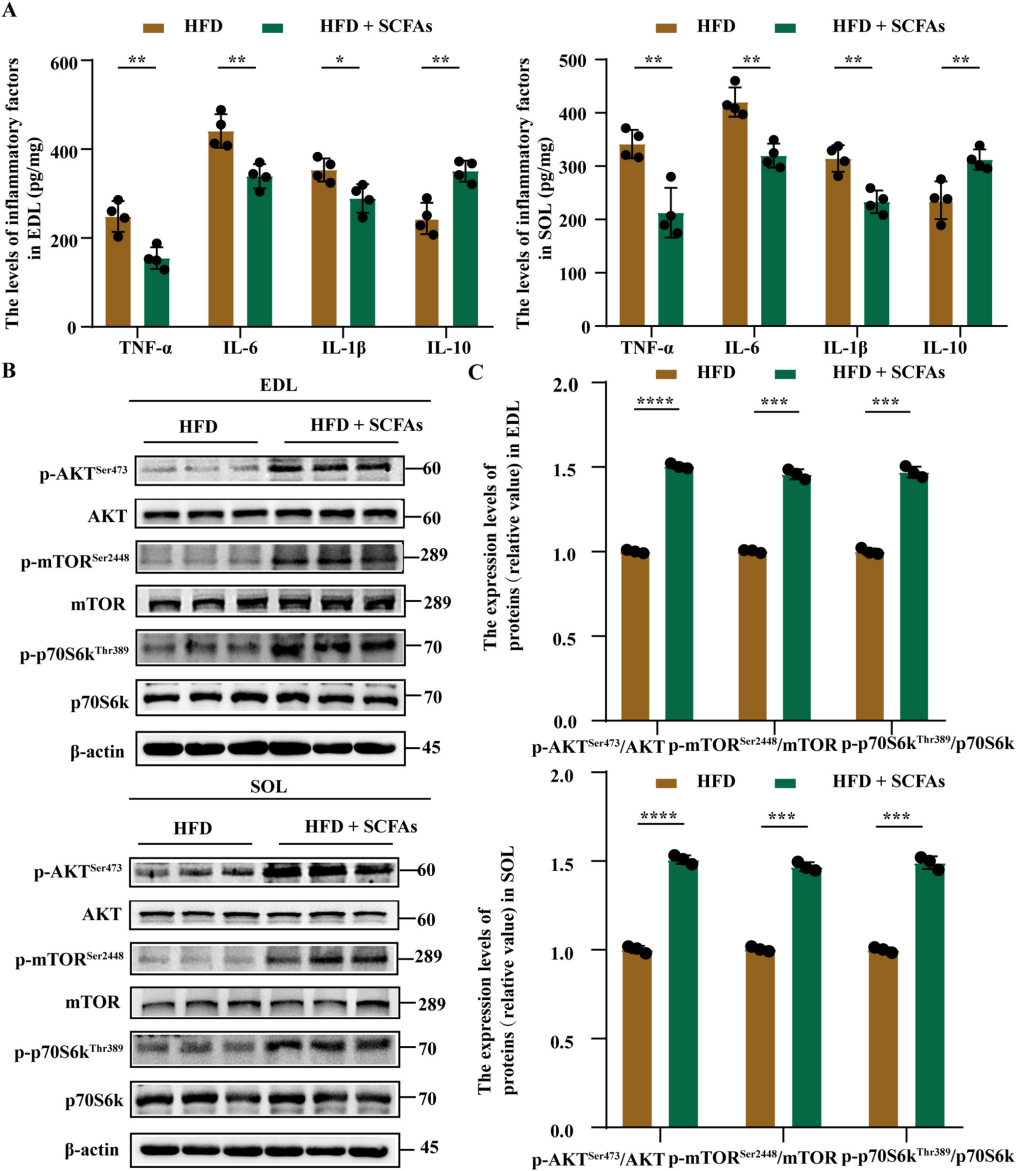
**SCFAs treatment reduces muscle inflammation and promotes muscle protein synthesis in HFD‐fed rats**. (A) The levels of tumour necrosis factor (TNF)‐α, interleukin (IL)‐1β, IL‐6, and IL‐10 in EDL and SOL muscles (*n* = 4 rats/group, repeated three times). (B) The protein bands of p‐Akt^473^, Akt, p‐mTOR^S2448^, mTOR, p‐p70S6k ^Thr389^, and p70S6k in EDL and SOL muscles (*n* = 3 rats/group, repeated three times). (C) Quantitative analysis of p‐Akt^473^/Akt, p‐mTOR^S2448^/mTOR, and p‐p70S6k ^Thr389^/p70S6k proteins in EDL and SOL muscles (*n* = 3 rats/group, repeated three times). Data were reported as mean ± SEM and analyzed by T‐test (A, C). *p < 0.05, **p < 0.01, ***p < 0.001 versus HFD‐fed rats.

SCFAs treatment was conducted to investigate the effect and mechanism of SCFAs in SO. The body weight of rats in the HFD + SCFAs group was lower than those in HFD‐fed rats, suggesting that SCFAs reduced HFD‐induced body weight gain (Figure S13A, *p* < 0.05). We found no significant differences in food intake and water intake between the two groups (Figure S13B, C, *p* > 0.05). HFD‐induced glucose and lipid metabolism disorders were inhibited by SCFAs (Figure S14A–E, all *p* < 0.05). HFD‐induced poor muscle mass, strength, function, and atrophy were antagonized by SCFAs (Figures S15 and S16, all *p* < 0.05). HFD‐induced morphological swelling and vacuolar degeneration of mitochondria, increased levels of reactive oxygen species (ROS), H_2_O_2_, and malondialdehyde (MDA), and a decreased level of superoxide dismutase (SOD) in EDL and SOL muscles were suppressed by SCFAs (Figure 7A–E, both *p* < 0.01). In addition, HFD‐induced increases in inflammation and decreases in the expression levels of p‐Akt^Ser473^/Akt, p‐mTOR^Ser2448^/mTOR, and p‐p70S6k^Thr389^/p70S6k in EDL and SOL muscles were mitigated by SCFAs (Figure 8A–C, all *p* < 0.05). Overall, MLT‐related increases in SCFAs alleviated the development of SO by suppressing metabolic disorders, reducing muscle oxidative stress and inflammation, and promoting protein synthesis via the AKT/mTOR/p70S6k signalling pathway.

## 4 Discussion

The serum levels of MLT decreased in SO patients and were closely associated with SO‐related parameters. MLT ameliorated the development of SO in HFD‐fed rats. It also regulated the dysbiosis of gut microbiota, promoted the production of SCFAs and repaired gut barrier impairment. The MLT‐related alterations in the gut microbiota and SCFAs were closely associated with SO. Faecal microbiota of MLT‐treated rats attenuated metabolic disorders and muscle atrophy in recipient rats. SCFAs treatments alleviated the development of SO by inhibiting metabolic disorders, muscle oxidative stress, and muscle inflammation and promoting muscle protein synthesis.

MLT decreases with age and plays a crucial role in the development of age‐related diseases [18]. MLT promoted muscle regeneration and muscle fibre hypertrophy in animal models [19, 20]. However, the association between MLT and sarcopenia or between MLT and SO has been rarely discussed in human studies. Lee et al. found an inverse association between urine MLT and sarcopenia in postmenopausal women, revealing the possible protective effect of MLT on sarcopenia [21]. In the present study, we found that serum MLT levels decreased in patients with SO. Moreover, the serum levels of MLT were negatively associated with BF% and positively associated with ASMI and handgrip strength. These findings revealed that MLT was associated with the development of SO.

Recently, much attention has been attracted on the effects of MLT on diseases via the gut microbiota. In this study, we attempted to elucidate the potential effect of MLT on SO from the perspective of gut microbiota and its metabolites. *Firmicutes* and *Bacteroidetes* are the major components of the gut microbiota [22]. We observed an increase in the relative abundance of *Firmicutes* and a decrease in the relative abundance of *Bacteroidetes* in HFD‐fed rats, consistent with microbiota composition reported in previous SO animal studies [7, 8]. However, these HFD‐induced alterations were alleviated by MLT, suggesting the potential antagonistic effect of MLT on SO via modulation of gut microbiota. Meanwhile, MLT increased the relative abundance of *Verrucomicrobiota* and its related *Akkermansiaceae* family and *Akkermansia* genus, which has been demonstrated to ameliorate SO in db/db mice [7]. Emerging evidence also indicated that the relative abundance of *Ruminococcus*, *Marvinbryantia* and *Lactobacillus* increased by MLT was closely associated with increases in muscle mass and muscle strength [23, 24]. In addition, the relative abundance of deleterious bacteria, such as *Eubacterium_nodatum_group* and *Lachnospiraceae_UCG−010*, reduced by MLT was closely related to systemic inflammation, which might aggravate the development of SO [25, 26]. The results of our association analysis showed that obesity‐related metabolic disorders and muscle atrophy were negatively associated with *Akkermansia*, *Ruminococcus*, *Marvinbryantia* and *Lactobacillus* and positively associated with *Eubacterium_nodatum_group* and *Lachnospiraceae_UCG−010*. Moreover, we found that faecal suspension from MLT‐treated rats promoted SCFAs production and improved metabolic disorders and muscle atrophy to a certain extent. Taken together, MLT inhibited the development of SO partially by regulating the gut microbiota.

SCFAs are the main microbial metabolites that play an important role in regulating oxidative stress and inflammatory responses [27]. *Akkermansia*, *Ruminococcus*, *Lactobacillus* and *Marvinbryantia* were related to the production of SCFAs [S11]. In the present study, we demonstrated a positive correlation between the abundance of these bacteria and SCFAs levels. Notably, MLT increased the abundance of these bacteria in faeces and the levels of SCFAs in faeces and serum. Epidemic research has supported the positive association of SCFAs with the skeletal muscle index [28, 29]. Animal studies also indicated that SCFAs treatment improved muscle mass and function [S9, 30]. In line with these studies, we found that SCFAs were positively associated with muscle mass, strength and function. Moreover, SCFAs treatment alleviated the reduction of muscle mass, strength and function in HFD‐fed rats. Nevertheless, the protective mechanism of SCFAs in muscle remains limited. Previous studies reported that SCFAs bound to their receptors, such as GPR41, GPR43 and GPR109A, which further inhibited the development of oxidative stress and inflammation [31, 32]. In line with these studies, we found that SCFAs treatments significantly reduced the levels of TNF‐α, IL‐6, IL‐1β, ROS, H_2_O_2_ and MDA in muscles, indicating that SCFAs treatments reduced muscle oxidative stress and inflammation. Muscle oxidative stress and inflammation are key factors leading to a decrease in protein synthesis and muscle atrophy [33, 34]. In this study, SCFAs treatments activated the AKT/mTOR/p70S6k signalling pathway in the EDL and SOL muscles of HFD‐fed rats, which was a typical signalling pathway involved in protein synthesis [35]. In addition, SCFAs modulated glucose and lipid metabolism, such as reducing glucose tolerance and dyslipidemia and enhancing insulin sensitivity [36]. These metabolic benefits might further promote muscle mass and function [16]. Hence, MLT increased the abundance of SCFAs‐related bacteria and the production of SCFAs, which inhibited metabolic disorders, muscle inflammation and muscle oxidative stress and improved protein synthesis, ultimately alleviating the development of SO.

The impairment of gut barrier integrity allows the translocation of deleterious metabolites into circulation, which results in systemic inflammation, affects protein synthesis and mitochondrial function, and ultimately facilitates the development of SO [37, 38]. Microbiota and its metabolites play a key role in maintaining gut barrier integrity. In the present study, HFD‐induced increases in *Eubacterium_nodatum_group*, and *Lachnospiraceae_UCG−010* might contribute to gut inflammation and disruption of gut barrier integrity [S12, S13]. However, MLT‐related increases in beneficial bacteria, such as *Akkermansia*, *Ruminococcus*, *Lactobacillus* and *Marvinbryantia*, restored the impaired gut barrier integrity by promoting the expression of tight junction proteins and the Muc‐2 protein [S14]. In addition, MLT increased the levels of SCFAs, which enhanced the expression of tight junction proteins and the Muc‐2 protein [S15]. These beneficial bacteria and SCFAs‐mediated integrity of gut barrier prevented the occurrence of systemic inflammation, obesity‐related metabolic diseases and muscle atrophy [S16]. These results indicated that MLT‐related increases in beneficial bacteria and SCFAs exerted a protective role in repairing gut barrier integrity, which might inhibit the development of SO.

To our knowledge, this study represented the first investigation to examine the protective effects of MLT on SO through the case‐control studies and SO rat models, providing novel insights into potential therapeutic strategies for mitigating SO caused by HFD. Our findings also demonstrated that MLT exerted protective effects against HFD‐induced SO through gut microbiota‐derived SCFAs, which reduced metabolic disorders, muscle oxidative stress and inflammation and promoted muscle protein synthesis via the AKT/mTOR/p70S6k signalling pathway. Nevertheless, several limitations should be acknowledged. First, the sample size of case‐control studies is relatively small. Further studies with larger sample sizes and adjustment for additional confounding variables are required for elucidating the association between serum MLT levels and SO. Second, further research is needed to investigate the detailed mechanisms by which SCFAs regulate the AKT/mTOR/p70S6k signalling pathway. Third, although our OTU‐based microbiota analysis revealed significant microbial community patterns associated with MLT, higher‐resolution methods (e.g., amplicon sequence variant (ASV) analysis) are needed to characterize taxon‐specific responses to MLT treatment. Finally, further studies are required for investigating the effects of MLT on SO in different genders.

## 5 Conclusion

MLT alleviated HFD‐induced SO by regulating the gut microbiota, promoting the production of SCFAs and repairing gut barrier integrity. FMT and SCFAs treatment further confirmed the crucial role of the gut microbiota and SCFAs in mediating the protective effects of MLT on SO. SCFAs treatments inhibited the development of SO by suppressing metabolic disorders, reducing muscle oxidative stress and inflammation, and increasing protein synthesis through the AKT/mTOR/p70S6k signalling pathway. Therefore, MLT might be an effective strategy for inhibiting the progression of SO.

## Ethics Statement

All human and animal studies were approved by the appropriate ethics committee and performed in accordance with the ethical standards laid down in the 1964 Declaration of Helsinki and its later amendments [39]. Informed consent was obtained from all patients for being included in the study.

## Conflicts of Interest

The authors declare no conflicts of interest.

## Supporting information


**Data S1** Supplementary Information.

**Data S2** Supplementary Information.

**Data S2** Supplementary Information.

**Table S1** The primer sequences of muscle atrophy‐related genes.
Table S2. Clinical characteristics of participants in this study.

## Data Availability

The sequencing data of the 16S rRNA gene sequencing are available in the Sequence Read Archive under project number PRJNA1246755. Additional data will be made available on request.
