# Supplementary material for: Melatonin Mitigates Sarcopenic Obesity via Microbiota and Short‐Chain Fatty Acids: Evidence From Epidemiologic and In Vivo Studies
Source: J Cachexia Sarcopenia Muscle. 2025 Jun 13;16(3):e13869. doi: 10.1002/jcsm.13869 (PMC12163512; doi:10.1002/jcsm.13869)
Supplement: Supplementary file 1 — Data S1 Supplementary Information. [file JCSM-16-e13869-s001.docx]

**Melatonin mitigates sarcopenic obesity via microbiota** **and short-chain fatty acids: Evidence from epidemiologic and *in vivo* studies**

**Journal of Cachexia Sarcopenia and Muscle**

Xiaoxing Mo^1^, Lihui Shen^1^, Xinyu Wang^1^, Wenqing Ni^2^, Linyan Li^1^, Lili Xia^1^, Hongjie Liu^1^, Ruijie Cheng^1^, Lin Wen^1^, Jian Xu ^2*^& Liegang Liu^1*^

^1^ Department of Nutrition and Food Hygiene, Hubei Key Laboratory of Food Nutrition and Safety, MOE Key Lab of Environment and Health, School of Public Health, Tongji Medical College, Huazhong University of Science and Technology, 13 Hangkong Road, Wuhan, 430030, China. [2024520214@hust.edu.cn](mailto:d202081565@hust.edu.cn), [M202275507@hust.edu.cn](mailto:M202275507@hust.edu.cn), [xywang_@hust.edu.cn,](mailto:xywang_@hust.edu.cn,) [d202181656@hust.edu.cn](mailto:d202181656@hust.edu.cn), [d202181612@hust.edu.cn,](mailto:d202181612@hust.edu.cn,) D201981405@hust.edu.cn, [d202381824@hust.edu.cn](mailto:d202381824@hust.edu.cn), [wenlin@hust.edu.cn](mailto:wenlin@hust.edu.cn), lgliu@mails.tjmu.edu.cn.

^2^ Department of Elderly Health Management, Shenzhen Center for Chronic Disease Control, Shenzhen, Guangdong, China. [wenqni@163.com](mailto:wenqni@163.com), anniexu73@126.com.

***Correspondence:**

Dr. Liegang Liu, Email: [lgliu@mails.tjmu.edu.cn](mailto:lgliu@mails.tjmu.edu.cn), Tel: +86 27 83650522, Fax: +86 27 83650522; Dr. Jian Xu, Email: [anniexu73@126.com](mailto:anniexu73@126.com)

**Supplementary methods**

**The levels of serum melatonin**

Blood samples were collected from the participants (*n* = 31 biologically independent samples per group) and the rats (*n* = 8 biologically independent samples per group) at 8:00 am–8:30 am after overnight fasting. The samples were then centrifuged at 3000 rpm for 15 min to obtain serum. Serum MLT levels were measured in triplicate using a commercial enzyme-linked immunosorbent assay kit (Enzo Life Sciences, Farmingdale, NY).

**Oral glucose tolerance test and Intraperitoneal insulin tolerance test**

Oral glucose tolerance test (OGTT) and Intraperitoneal insulin tolerance test (ipITT) were used to evaluate glucose tolerance and insulin sensitivity, respectively (*n* = 8 biologically independent samples per group). In OGTT, rats were orally administered with 2 g/kg 50% D-glucose solution (Sigma–Aldrich, St. Louis, MO, USA) after overnight fasting. In ipITT, rats received 0.75 IU/kg insulin (Novo Nordisk, Denmark) through intraperitoneal injection after 6 h of fasting. Blood glucose was detected using a glucometer (Roche, Grenzach-Wyhlen, Germany) at 0, 15, 30, 60, 90, and 120 mins. The blood glucose of each rat was tested thrice, and the three measured values were recorded and averaged. Area under the curve (AUC) of blood glucose was calculated.

**Magnetic resonance imaging**

The muscle volumes of rats were evaluated using magnetic resonance imaging (MRI) (*n* = 3 biologically independent samples per group) [1]. In brief, rats were anesthetized with 1%–2% isoflurane and scanned using a Siemens 3T scanner (Siemens MAGNETOM, Germany) with a small animal coil. T1-weighted images were applied with the following parameters: 1060/15 ms TR/TE, 64 mm FOV read, 2 mm section thickness, 15 number of slices, and 0.2 mm × 0.2 mm × 2.0 mm voxel size. Muscle volume was calculated using Sante DICOM Editor.

**Behavior tests**

Behavior tests including grip strength test, rotarod test, and exhaustive running test were used to evaluate muscle strength and function (*n* = 8 biologically independent samples per group) [1].

1. Grip strength test

Rats were placed on a grid, and their tails were pulled back. The maximum grip strength was obtained when the limbs of rats were released from the grid. The grip strength of each rat was tested thrice, and the three measured values were recorded and averaged

1. Rotarod test

Rats were placed on a rotating rod, and the speed of the rod gradually increased from 0 rpm to 50 rpm within 5 min. The time and speed of rats on the rotating rod were recorded when they were released from that rod.

1. Exhaustive running test

Rats were acclimated to the treadmill at 0 m/s for a day and then acclimated to the treadmill at 10 m/s on the next day. The speed of the treadmill started from 10 cm/s and accelerated by 5 cm/s, accompanied with an increase in slope angle at 2°/3 min on the third day. The time and total distance of rats were recorded when they were unable to return to the treadmill over 20 s.

**16S rRNA gene sequencing**

16S rRNA gene sequencing was conducted to clarify the composition of the gut microbiota (*n* = 8 biologically independent samples per group) [2]. Each rat was placed in a sterile cage until defecation. Fresh fecal pellets were collected with sterile forceps, placed in sterile centrifuge tubes, and rapidly frozen in liquid nitrogen. Fecal DNA was extracted using a DNA kit, quantified using a Qubit 2.0 fluorometer, amplified using primers (V3–V4 region), and sequenced on an Illumina MiSeq platform (Illumina, San Diego, CA, USA). Raw reads were preprocessed using the Quantitative Insights into Microbial Ecology software package (QIIME, Boulder, CO, USA, V.1.9.1). Operational taxonomic units (OTUs) with 97% similarity of reads were annotated on the Silva Database. Non-metric multidimensional scaling (NMDS) based on the unweighted UniFrac distance algorithm was used to assess β-diversity. The differences in the compositions of gut microbiota among the three groups were analyzed using the Kruskal-Wallis test, followed by Dunn's post hoc test for multiple comparisons. *P* values were adjusted for false discovery rate (FDR) using the Benjamini-Hochberg method, with adjusted *P* < 0.05 considered statistically significant.

**Measurement of short-chain fatty acids**

The levels of short-chain fatty acids (SCFAs) in feces and serum were detected using gas chromatography-mass spectrometry (GC-MS) as previously described (*n* = 8 biologically independent samples per group) [3]. The serum (100 μL) was mixed with 200 μL of acetone:water (2:1, v/v) and centrifuged at 14,000 g at 4 ℃ for 10 min. The supernatant (180 μL) was collected and derivatized with 60 μL of internal standard (0.1 mM 2-ethylbutyric acid), 100 μL of 100 mM 2,3,4,5,6-pentafluorobenzyl bromide (PFBBr) acetone solution, 140 μL of acetone, and 60 μL of phosphate-buffered saline (PBS), followed by incubation at 60 °C for 2 hours. After cooling down, the mixture was extracted with 500 μL hexane:water (3:2, v/v), and the upper hexane was collected for GC-MS analysis. Chromatographic separation was performed on an Agilent HP-5ms column (30 m × 0.25 mm × 0.25 μm) with He carrier gas at a flowrate of 1.5 mL/min. The temperature of the column gradually increased from 80 °C to 158 °C at 10 °C /min, from 158 °C to 160 °C at 3 °C/min, and from 160 °C to 220 °C at 20 °C/min. Feces were homogenized with PBS and centrifuged at 12,000 rpm for 10 min. The supernatant was collected and subjected to the same derivatization and analytical procedures as described for serum samples. A calibration curve was generated using SCFAs standards (5, 2, 1, 0.4, 0.1, 0.025, 0.01, and 0 mM). The detection limit was 1 μM, with a precision of <5% (intra-day relative standard deviation) and <10% (inter-day relative standard deviation). The levels of SCFAs were quantified using Agilent ChemStation data analysis software (version F.01.01).

**Serum** **lipid levels**

The levels of serum lipids, including total cholesterol (TC), triglyceride (TG), high-density lipoprotein cholesterol (HDL-C), and low-density lipoprotein cholesterol (LDL-C), were measured using commercial kits (Nanjing Jiancheng, Nanjing, China). Each group contained 8 biologically independent replicates, and the experiment was repeated three times.

**Measurement of inflammatory factors**

Inflammatory factors, including tumor necrosis factor (TNF)-α, interleukin (IL)-1β, IL-6, and IL-10, were detected in extensor digitorum longus (EDL) and soleus (SOL) muscles. These muscles were homogenized with PBS and centrifuged at 12,000 rpm for 10 min to collect the supernatant. Inflammatory factors in the supernatant were detected by ELISA kits according to the manufacturer’s guidelines (Elabscience, Wuhan, China). Each group contained 4 biologically independent replicates, and the experiment was repeated three times.

**Reactive oxygen species quantification**

The frozen sections of EDL and SOL muscles were prepared and stained with dihydroethidium (DHE, Cat #D7008, Sigma) to assess the levels of reactive oxygen species (ROS) (*n* = 4 biologically independent samples per group). The fluorescence intensities of ROS were quantified by Image J software.

**Oxidative stress marker measurements**

EDL and SOL muscles were homogenized and centrifuged at 12000 rpm for 15 min to obtain the supernatant. The content of H_2_O_2_ and the activities of superoxide dismutase (SOD) and malondialdehyde (MDA) were measured by commercial ELISA kits (Solarbio, China). Each group contained 4 biologically independent replicates, and the experiment was repeated three times.

**Transmission electron micrographs**

EDL and SOL muscles were dissected immediately and fixed in 2.5% glutaraldehyde at 4 °C overnight (*n* = 3 biologically independent samples per group). The tissues were fixed in 1% OsO4, stained with 1% uranylacetate, and embedded in Epon. After ultramicrotomy, the slices were stained with uranyl acetate and lead citrate, and the mitochondria were captured. The size of the mitochondria was quantified by Image J software.

**Histological analysis**

Paraffin-embedded EDL and SOL muscles were prepared, and cross-sectional 7–8 μm slices of EDL and SOL muscles were stained with hematoxylin and eosin (H&E) to evaluate histopathological changes (*n* = 4 biologically independent samples per group). The cross-sectional area (CSA) of EDL and SOL muscles was analyzed using Image J software.

**Immunofluorescence staining**

Paraffin-embedded colon tissues were prepared (*n* = 4 biologically independent samples per group), and cross-sectional 7–8 μm slices of colon tissues were incubated with mucin (Muc)-2 (Cat# NBP2-66961, 1:100, Novus) primary antibodies and then with Alexa Fluor 488-labeled secondary antibody (Cat# Z25306, 1:300, Thermo Fisher Scientific) and DAPI (Cat# D9542, 1:10000, Sigma). Fluorescence intensity was quantified by Image J software.

**Quantitative real-time PCR**

Total RNA was extracted from EDL and SOL muscles by using TRIzol reagent (Invitrogen, CA, USA). RNA was reverse transcribed into cDNA by using a cDNA synthesis kit (Invitrogen, CA, USA), and cDNA was analyzed by qRT-PCR using SYBR Green® Premix Ex Taq (Invitrogen, CA, USA) kit. The primer sequences of muscle atrophy-related genes were shown in Table S1. Each group contained 4 biologically independent replicates, and the experiment was repeated three times.

**Western blot**

Total proteins of EDL and SOL muscles were lysed in RIPA lysis buffer and quantified using BCA kits. An equal amount of protein was loaded on SDS-PAGE gel and transferred onto nitrocellulose membranes. The membranes were blocked by 5% milk and incubated with primary antibodies as follows: claudin-1 (Cat # ab15098, 1:1000, Abcam), occludin (Cat# ab216327, 1:1000, Abcam), zonula occluden (Zo)-1 (Cat# 40-2200, 1:1000, Thermo Scientific), p-Akt^Ser^473 (Cat#9271, 1:1000, Cell Signaling Technology), Akt (Cat#9272, 1:1000, Cell Signaling Technology), p-mTOR^Ser2448^ (Cat#2971, 1:1000, Cell Signaling Technology), mTOR (Cat#2983, 1:1000, Cell Signaling Technology), p-p70S6k^Thr389^ (Cat# #9205, 1:1000, Cell Signaling Technology), and p70S6k (Cat#2708, 1:1000, Cell Signaling Technology). After washing, the membranes were incubated with secondary antibodies and detected using a Box-HR-E-M imaging system (SYNGENE). Protein quantification was conducted using Image J software. Each group contained 3 biologically independent replicates, and the experiment was repeated three times.

**References**

1. Mo X, Cheng R, Shen L, Sun Y, Wang P, Jiang G, et al. High-fat diet induces sarcopenic obesity in natural aging rats through the gut-trimethylamine N-oxide-muscle axis. J Adv Res. 2025;70:405-422.

2. Liu J, Liu Y, Huang C, He C, Yang T, Ren R, et al. Quercetin-Driven Akkermansia Muciniphila Alleviates Obesity by Modulating Bile Acid Metabolism via an ILA/m(6)A/CYP8B1 Signaling. Adv Sci (Weinh). 2025;12(12):e2412865.

3. Xu M, Mo X, Huang H, Chen X, Liu H, Peng Z, et al. Yeast beta-glucan alleviates cognitive deficit by regulating gut microbiota and metabolites in Abeta(1)(-)(42)-induced AD-like mice. Int J Biol Macromol. 2020;161:258-270.
