# Supplementary material for: Melatonin Mitigates Sarcopenic Obesity via Microbiota and Short‐Chain Fatty Acids: Evidence From Epidemiologic and In Vivo Studies
Source: J Cachexia Sarcopenia Muscle. 2025 Jun 13;16(3):e13869. doi: 10.1002/jcsm.13869 (PMC12163512; doi:10.1002/jcsm.13869)
Supplement: Supplementary file 2 — Data S2 Supplementary Information. [file JCSM-16-e13869-s002.docx]

**Melatonin mitigates sarcopenic obesity via microbiota** **and short-chain fatty acids: Evidence from epidemiologic and *in vivo* studies**

**Journal of Cachexia Sarcopenia and Muscle**

Xiaoxing Mo^1^, Lihui Shen^1^, Xinyu Wang^1^, Wenqing Ni^2^, Linyan Li^1^, Lili Xia^1^, Hongjie Liu^1^, Ruijie Cheng^1^, Lin Wen^1^, Jian Xu ^2*^& Liegang Liu^1*^

^1^ Department of Nutrition and Food Hygiene, Hubei Key Laboratory of Food Nutrition and Safety, MOE Key Lab of Environment and Health, School of Public Health, Tongji Medical College, Huazhong University of Science and Technology, 13 Hangkong Road, Wuhan, 430030, China. [2024520214@hust.edu.cn](mailto:d202081565@hust.edu.cn), [M202275507@hust.edu.cn](mailto:M202275507@hust.edu.cn), [xywang_@hust.edu.cn,](mailto:xywang_@hust.edu.cn,) [d202181656@hust.edu.cn](mailto:d202181656@hust.edu.cn), [d202181612@hust.edu.cn,](mailto:d202181612@hust.edu.cn,) D201981405@hust.edu.cn, [d202381824@hust.edu.cn](mailto:d202381824@hust.edu.cn), [wenlin@hust.edu.cn](mailto:wenlin@hust.edu.cn), lgliu@mails.tjmu.edu.cn.

^2^ Department of Elderly Health Management, Shenzhen Center for Chronic Disease Control, Shenzhen, Guangdong, China. [wenqni@163.com](mailto:wenqni@163.com), anniexu73@126.com.

***Correspondence:**

Dr. Liegang Liu, Email: [lgliu@mails.tjmu.edu.cn](mailto:lgliu@mails.tjmu.edu.cn), Tel: +86 27 83650522, Fax: +86 27 83650522; Dr. Jian Xu, Email: [anniexu73@126.com](mailto:anniexu73@126.com).

**Supplementary figures**


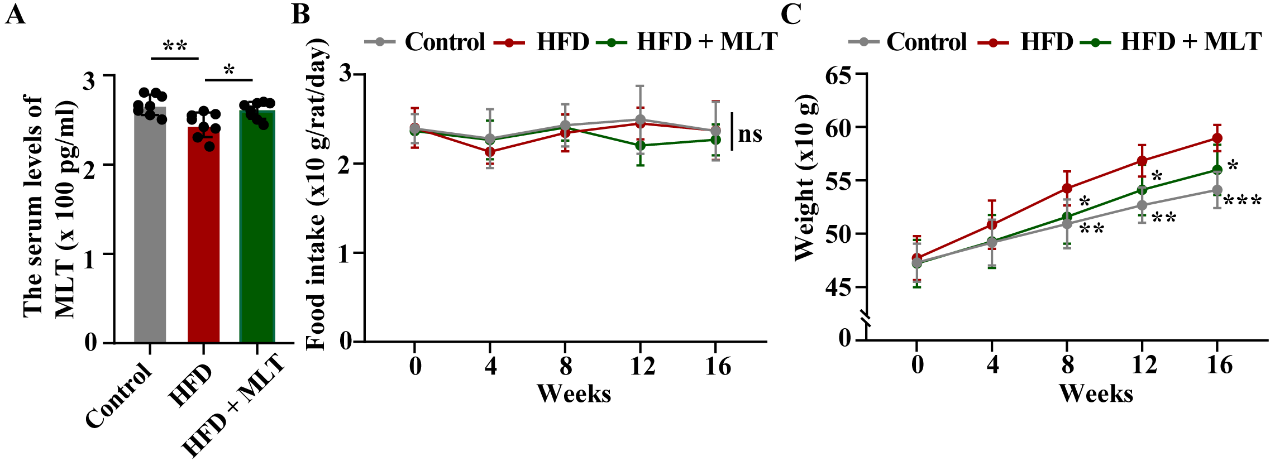


**Figure S1 Effects of MLT intervention on serum MLT levels, food intake, and body weight in HFD-fed rats.** (A) The levels of MLT in serum (*n* = 8 rats/ group, repeated three times). (B) Food intake (*n* = 8 rats/group). (C) Body weight (*n* = 8 rats/group). Data were reported as mean ± s.e.m and analyzed by one-way ANOVA, followed by Tukey’s multiple comparisons test (A–C). **P*<0.05, ***P*<0.01, ****P*<0.001 versus HFD-fed rats. ns: not significant.


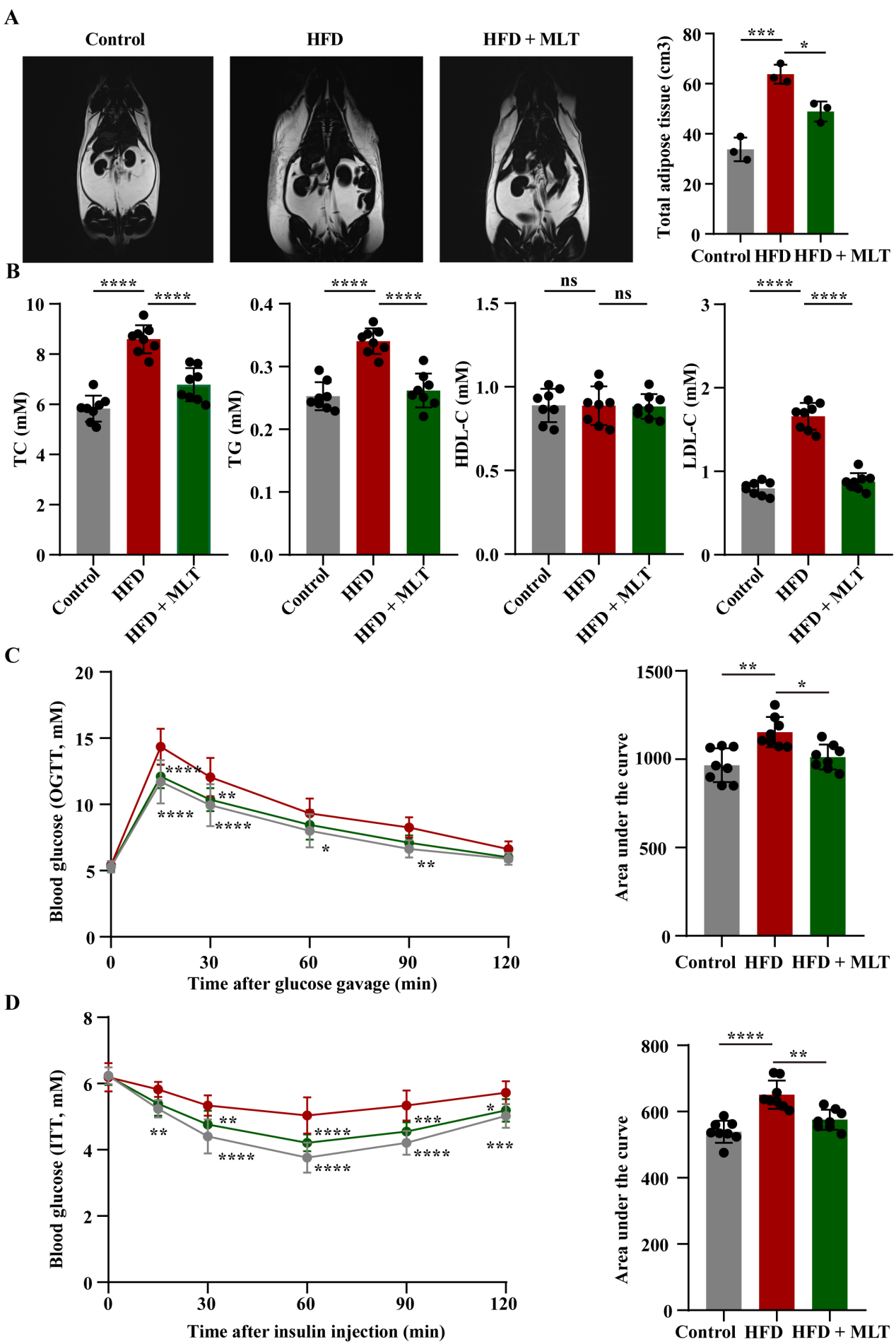


**Figure S2 MLT alleviates obesity-related metabolic disorders in HFD-fed rats.** (A) The representative images of adipose deposition examined by magnetic resonance imaging (MRI), and quantitative analysis of fat volume (*n* = 3 rats/group). (B) The levels of total cholesterol (TC), triglyceride (TG), high-density lipoprotein cholesterol (HDL-C), and low-density lipoprotein cholesterol (LDL-C) in serum (*n* = 8 rats/group, repeated three times). (C) The blood glucose at 0, 15, 30, 60, 90, and 120 mins after oral administrated with D-glucose solution, and the area under the curve values (AUC) value of blood glucose in oral glucose tolerance test (OGTT) (*n* = 8 rats/group). (D) The blood glucose at 0, 15, 30, 60, 90, and 120 mins after intraperitoneal injection with insulin, and the AUC value of blood glucose in intraperitoneal insulin tolerance test (ipITT) (*n* = 8 rats/group). Data were reported as mean ± s.e.m and analyzed by one-way ANOVA, followed by Tukey’s multiple comparisons test (B–D). **P*<0.05, ***P*<0.01, ****P*<0.001, *****P*<0.0001 versus HFD-fed rats. ns: not significant.

**
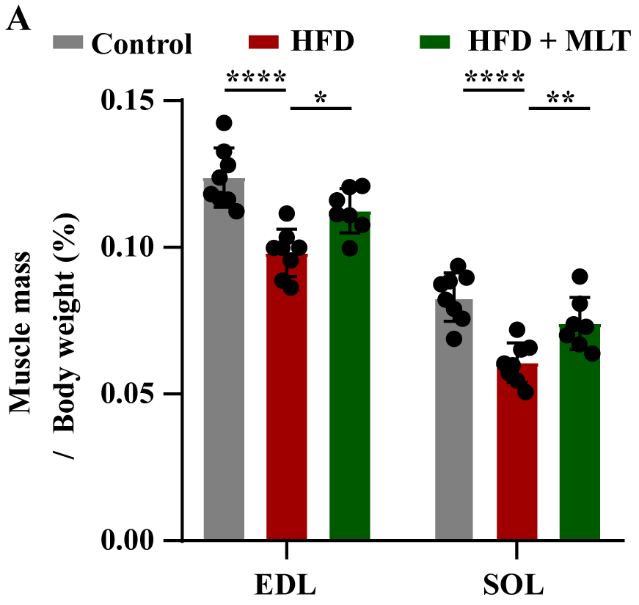
**

**Figure S3 MLT treatment increases muscle mass in HFD-fed rats.** (A) The weights of extensor digitorum longus (EDL) and soleus (SOL) muscles (*n* = 8 rats/group). Data were reported as mean ± s.e.m and analyzed by one-way ANOVA, followed by Tukey’s multiple comparisons test (A). **P*<0.05, ***P*<0.01, *****P*<0.0001 versus HFD-fed rats.


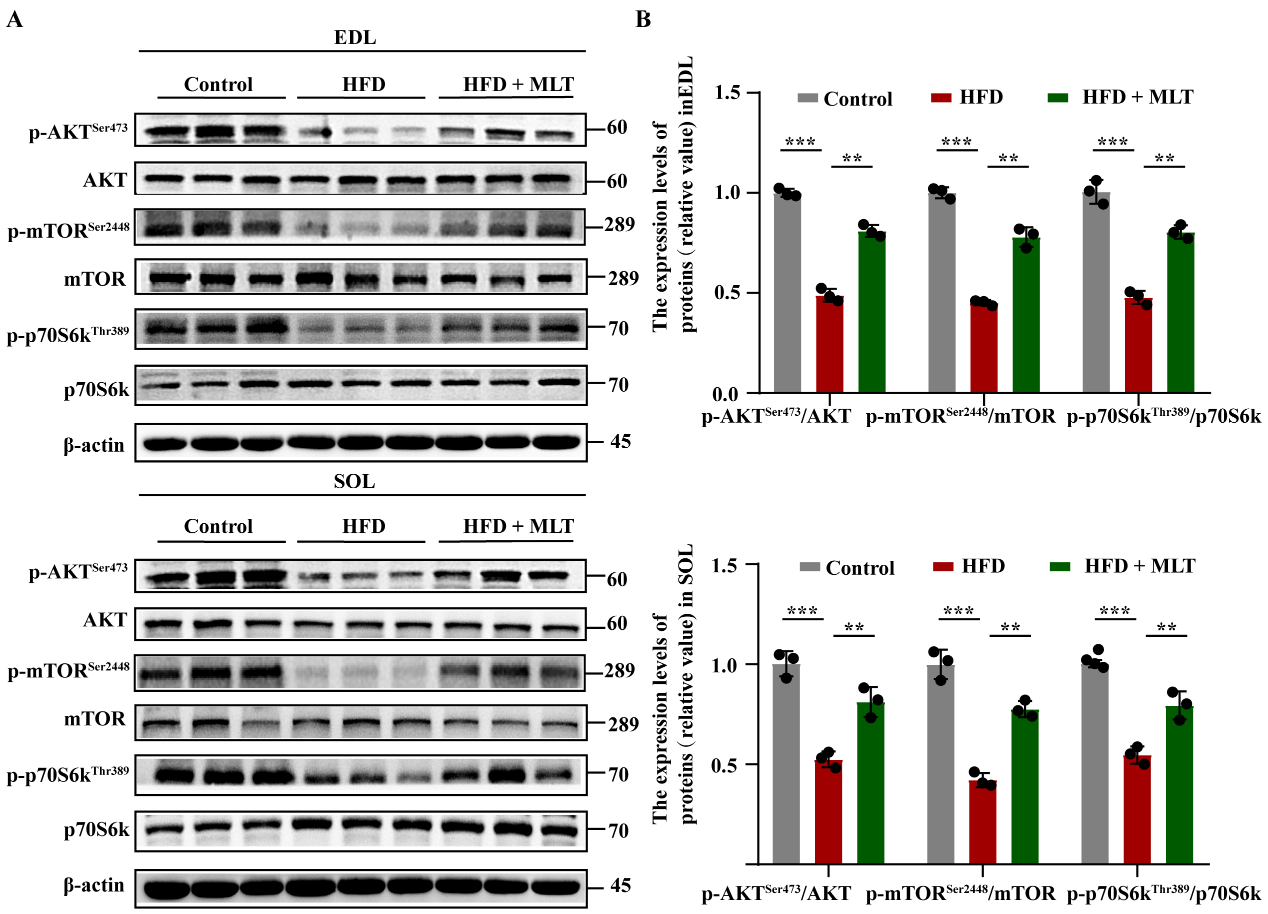


**Figure S4 MLT promotes protein synthesis in HFD-fed rats.** (A) The protein bands of p-Akt^473^, Akt, p-mTOR^S2448^, mTOR, p-p70S6k^Thr389^, and p70S6k (*n* = 3 rats/group, repeated three times). (B) Quantitative analysis of p-Akt^473^/Akt, p-mTOR^S2448^/mTOR, and p-p70S6k ^Thr389^/p70S6k proteins (*n* = 3 rats/group, repeated three times). Data were reported as mean ± s.e.m and analyzed by one-way ANOVA, followed by Tukey’s multiple comparisons test (B). ***P*<0.01, ****P*<0.001 versus HFD-fed rats.


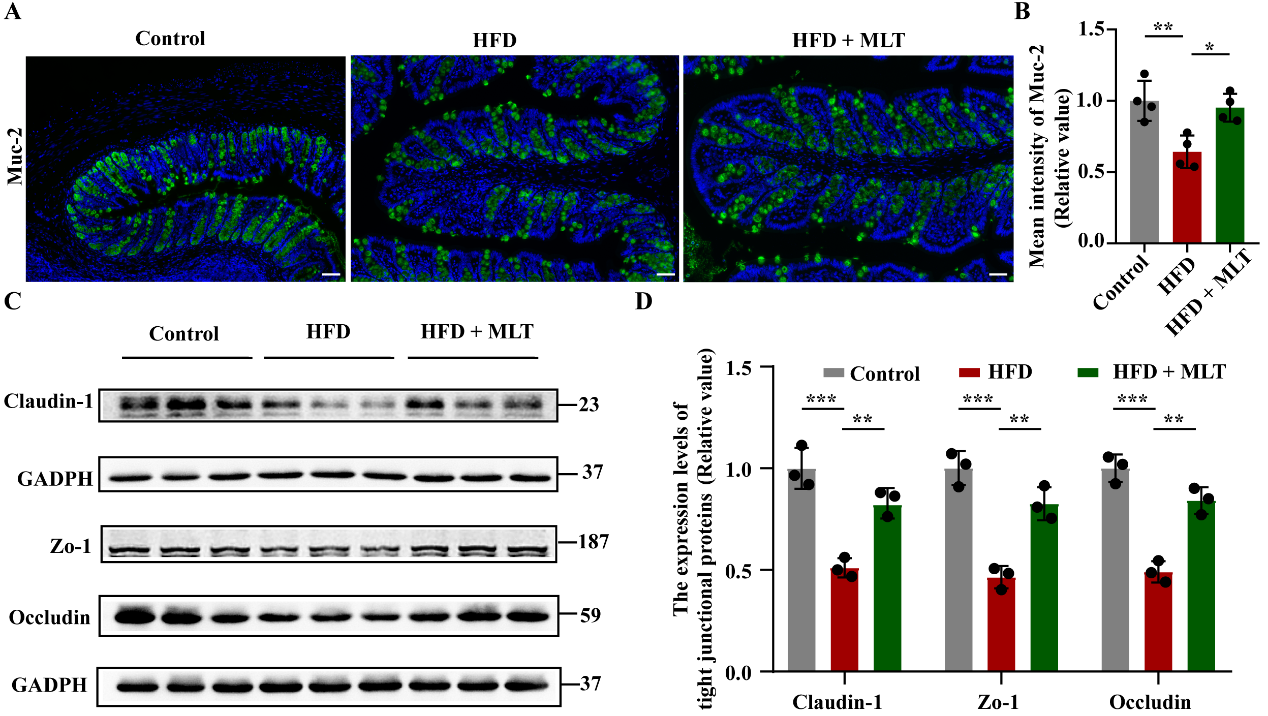


**Figure S5 MLT repaired the impairment of gut barrier integrity in HFD-fed rats.** (A) The representative images of immunofluorescence staining of mucin (Muc)-2 (*n* = 4 rats/group). (B) Quantitative analysis of Muc-2 fluorescence intensity (*n* = 4 rats/group). (C) The protein bands of Claudin-1, zonula occluden (Zo) -1, and Occludin (*n* = 3 rats/group, repeated three times). (D) Quantitative analysis of Claudin-1, Zo-1, and Occludin proteins (*n* = 3 rats/group, repeated three times). Data were reported as mean ± s.e.m and one-way ANOVA, followed by Tukey’s multiple comparisons test (B, D). **P*<0.05, ***P*<0.01, ****P*<0.001 versus HFD-fed rats.


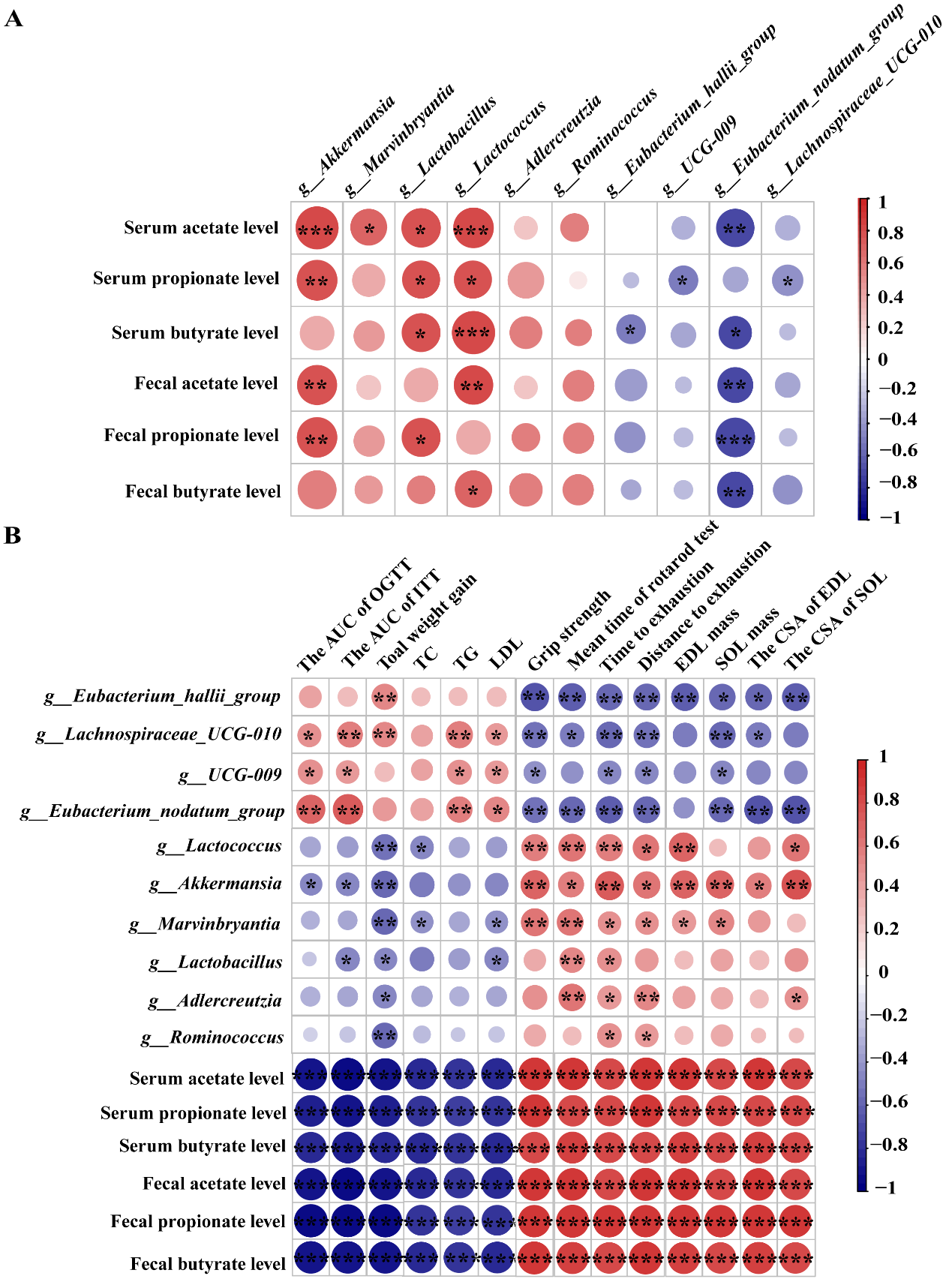


**Figure S6 Correlation heatmap analysis of microbiota, short-chain fatty acids, and SO.** (A) Correlation analysis between gut microbiota and short-chain fatty acids (SCFAs). (B) Correlation analysis of gut microbiota, SCFAs, and SO. Correlation analysis was assessed by Spearman's correlation analysis. *P* values were adjusted for false discovery rate (FDR) using the Benjamini-Hochberg method, with adjusted *P*<0.05 considered statistically significant. **P*<0.05, ***P*<0.01, ****P*<0.001.

**
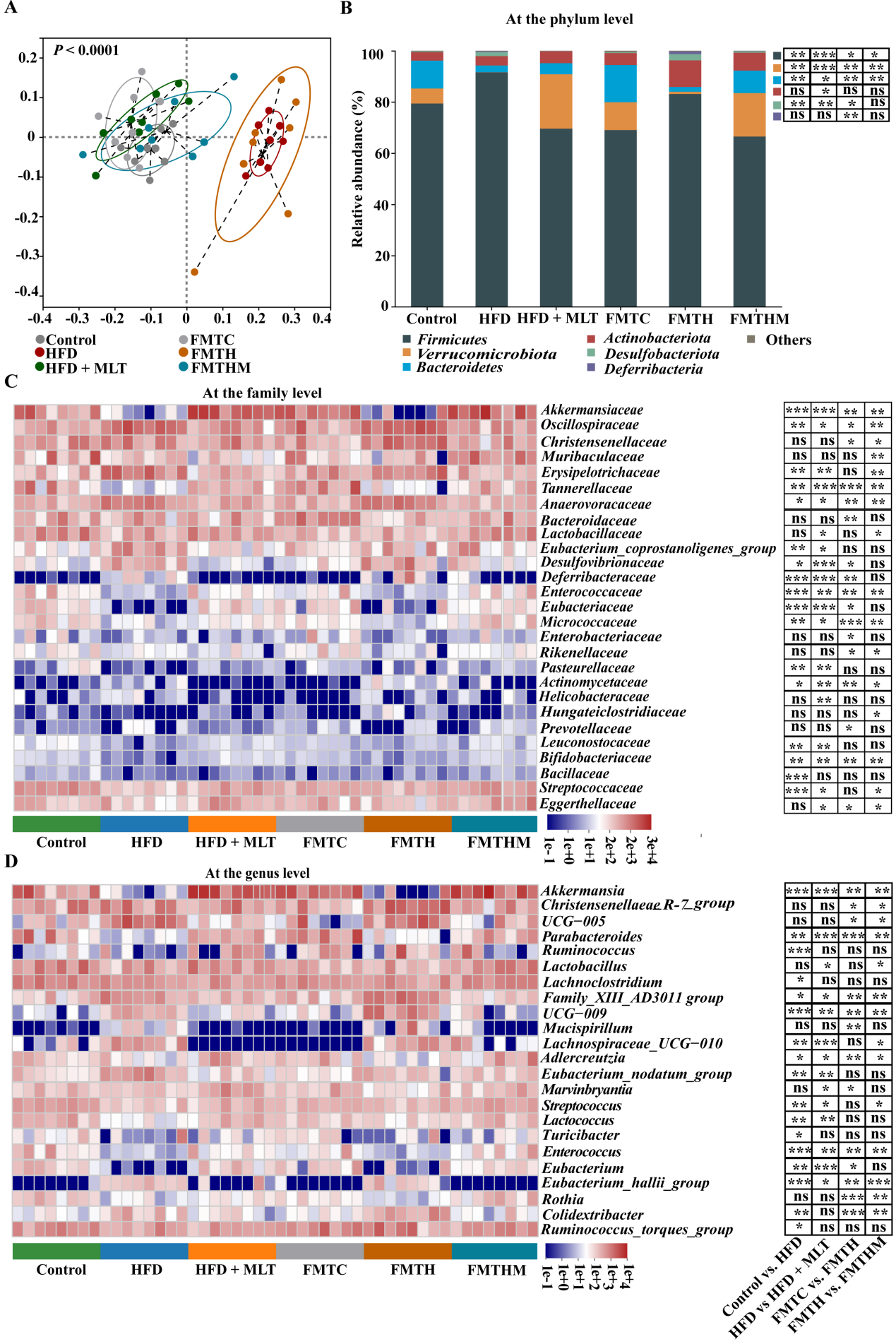
**

**Figure S7 The effect of fecal microbiota transplantation on the gut microbiota in recipient rats.** To enable direct comparison, Figure S7 presented the gut microbiota composition of both donor and recipient rats (donor data partially duplicated from Figure 4). (A) The β-diversity of gut microbiota based on non-metric multidimensional scaling (NMDS) analysis (*n* = 8 rats/group). (B) The relative abundance of gut microbiota at the phylum level (*n* = 8 rats/group). (C) Heatmap of gut microbiota at the family level (*n* = 8 rats/group). (D) Heatmap of gut microbiota at the genus level (*n* = 8 rats/group). Data were analyzed by Kruskal–Wallis test, followed by Dunn’s Multiple Comparison post-test (B–D). *P*-values were adjusted for FDR using the Benjamini-Hochberg method, with adjusted *P*<0.05 considered statistically significant. **P*<0.05, ***P*<0.01, ****P*<0.001 versus the recipient rats received fecal suspension of HFD-fed rats. ns: not significant.

**
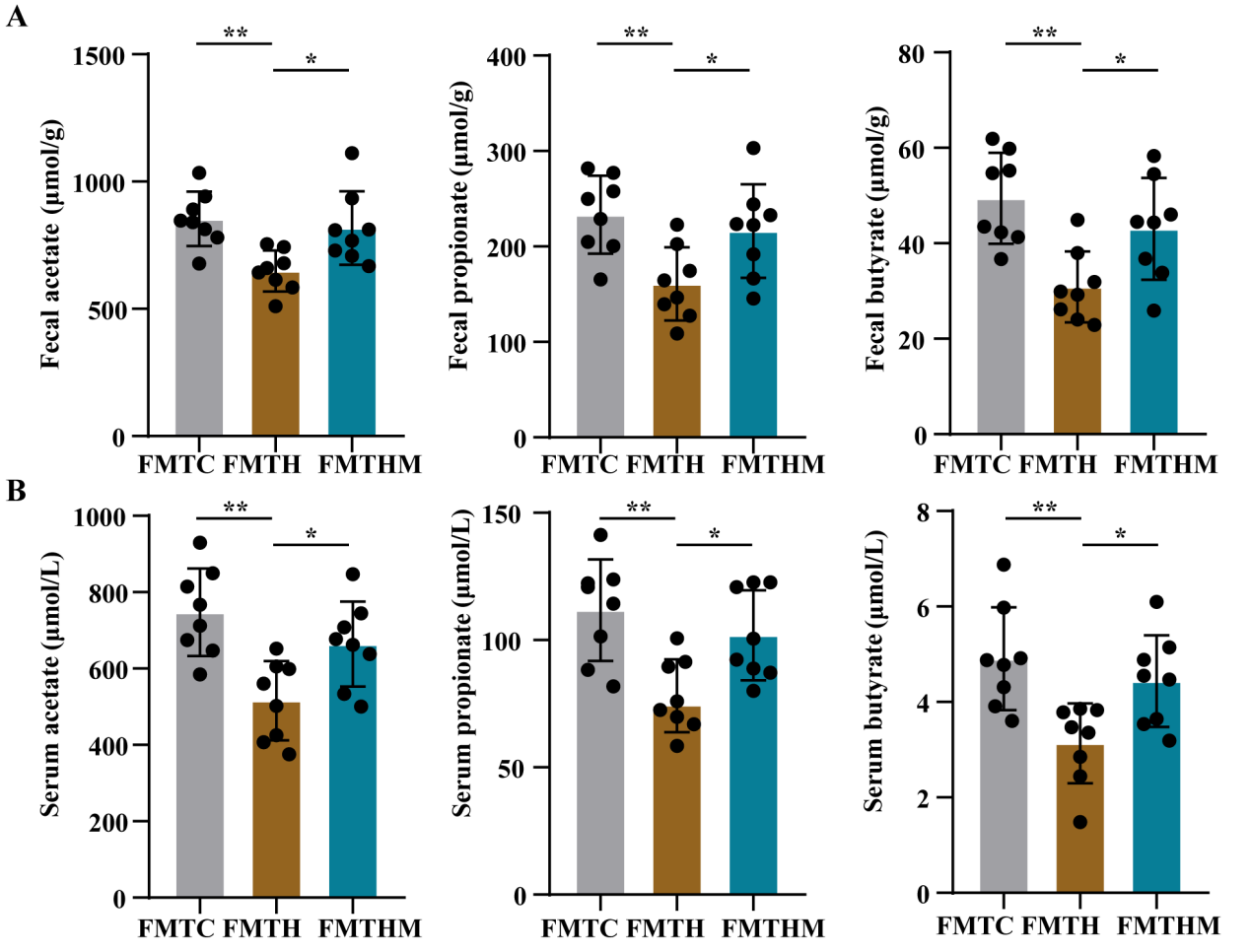
**

**Figure S8 Fecal suspension of MLT-treated rats promotes the production of SCFAs in recipient rats.** (A) The levels of acetate, propionate, and butyrate in feces (*n* = 8 rats/group). (B) The levels of acetate, propionate, and butyrate in serum (*n* = 8 rats/group). Data were reported as mean ± s.e.m and analyzed by one-way ANOVA, followed by Tukey’s multiple comparisons test (A, B). **P*<0.05, ***P*<0.01 versus the recipient rats received fecal suspension of HFD-fed rats.


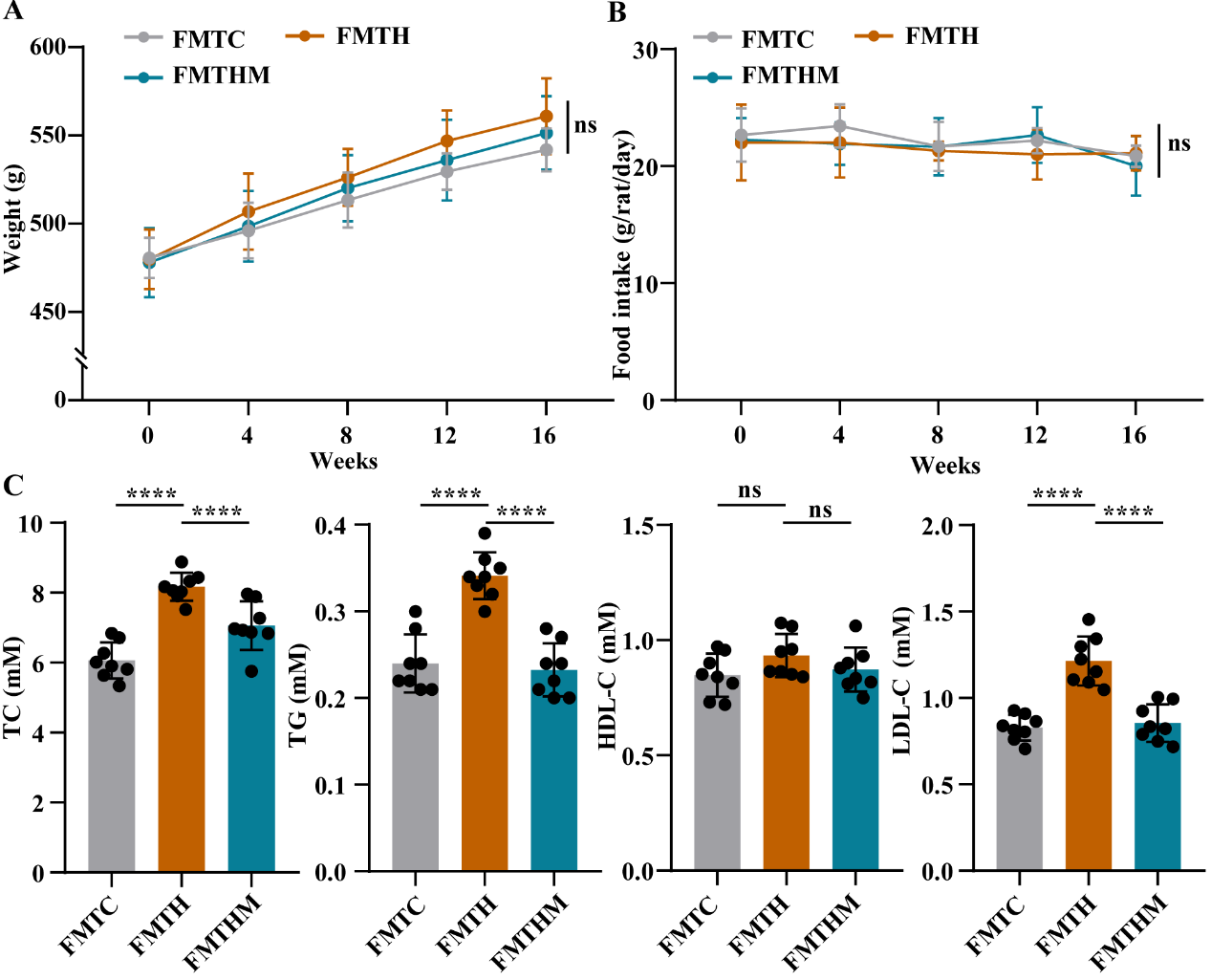


**Figure S9 The effects of fecal microbiota transplantation on body weight, food intake, and blood lipids in recipient rats.** (A) Body weight (*n* = 8 rats/group). (B) Food intake (*n* = 8 rats/group). (C) The levels of TC, TG, HDL-C, and LDL-C in serum (*n* = 8 rats /group, repeated three times). Data were reported as mean ± s.e.m and analyzed by one-way ANOVA, followed by Tukey’s multiple comparisons test (A–C). *****P*<0.0001 versus the recipient rats received fecal suspension of HFD-fed rats. ns: not significant.


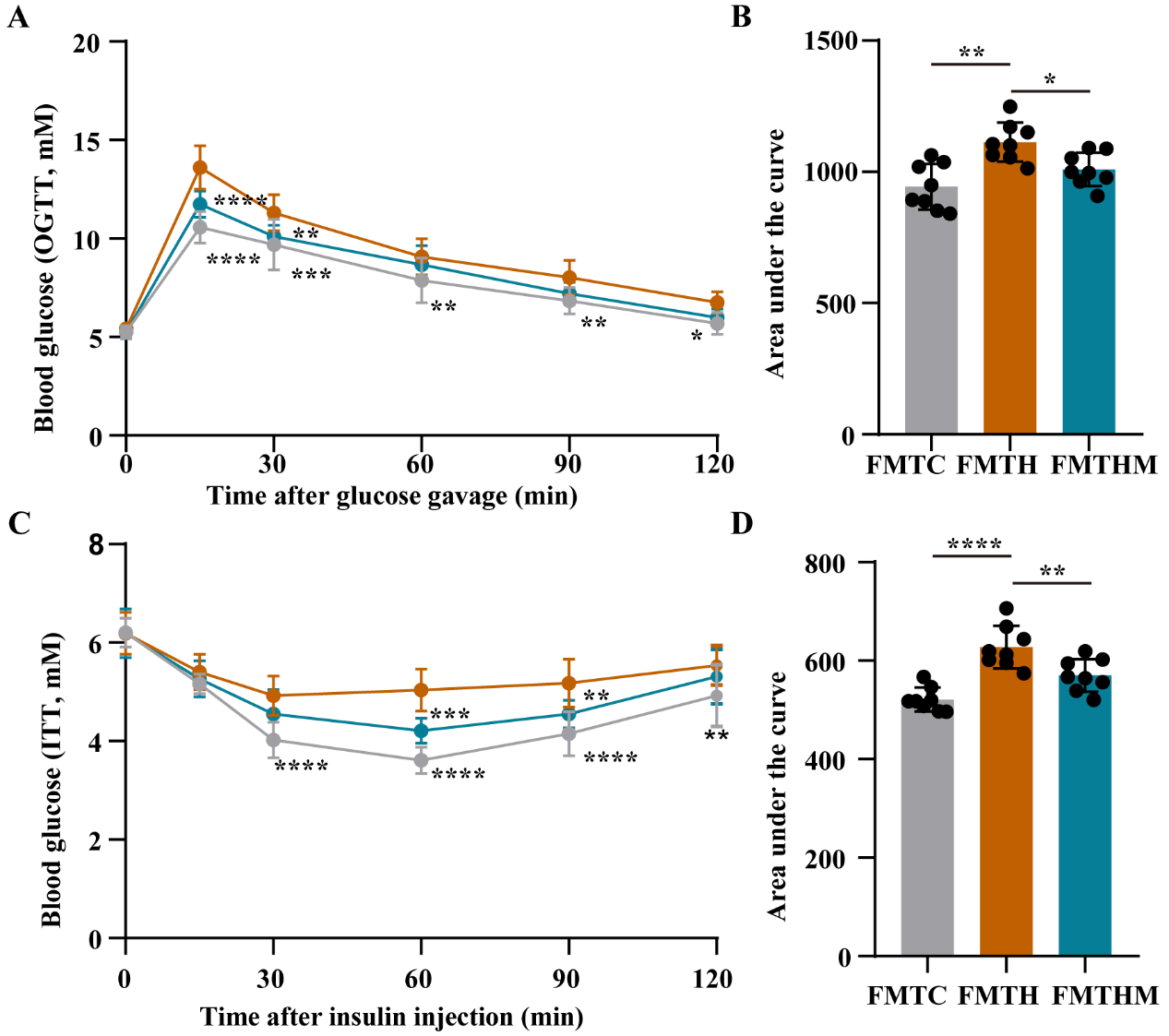


**Figure S10 Fecal suspension of MLT-treated rats regulates glucose metabolism disorders in recipient rats.** (A) The blood glucose at 0, 15, 30, 60, 90, and 120 mins after oral administrated with D-glucose solution (*n* = 8 rats/group). (B) The AUC value of blood glucose in OGTT (*n* = 8 rats/group). (C) The blood glucose at 0, 15, 30, 60, 90, and 120 mins after intraperitoneal injection with insulin (*n* = 8 rats/group). (B) The AUC value of blood glucose in ipITT (*n* = 8 rats/group). Data were reported as mean ± s.e.m and analyzed by one-way ANOVA (A–D), followed by Tukey’s multiple comparisons test. **P*<0.05, ***P*<0.01, ****P*<0.001, *****P*<0.0001 versus the recipient rats received fecal suspension of HFD-fed rats.


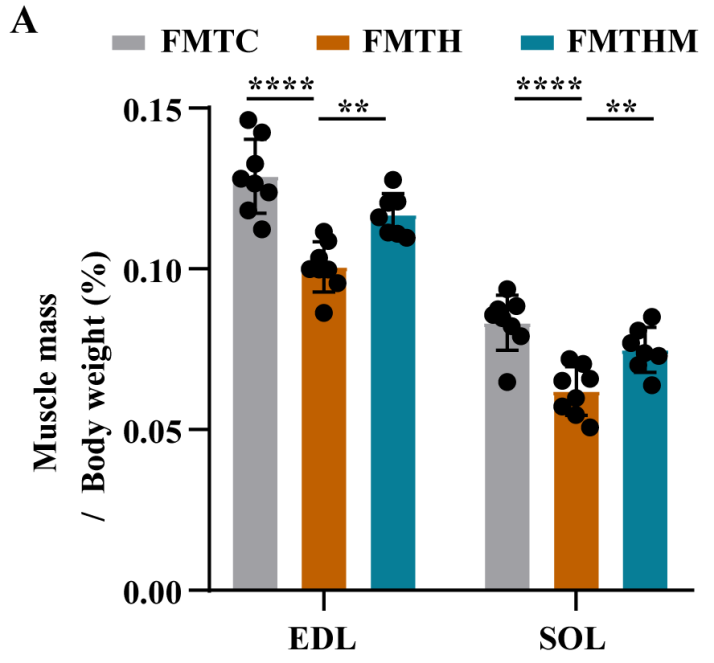


**Figure S11 Fecal suspension of MLT-treated rats increases muscle mass in receipt rats.** (A) The weights of EDL and SOL muscles (*n* = 8 rats/group). Data were reported as mean ± s.e.m and analyzed by one-way ANOVA, followed by Tukey’s multiple comparisons test. ***P*<0.01, *****P*<0.0001 versus the recipient rats received fecal suspension of HFD-fed rats.


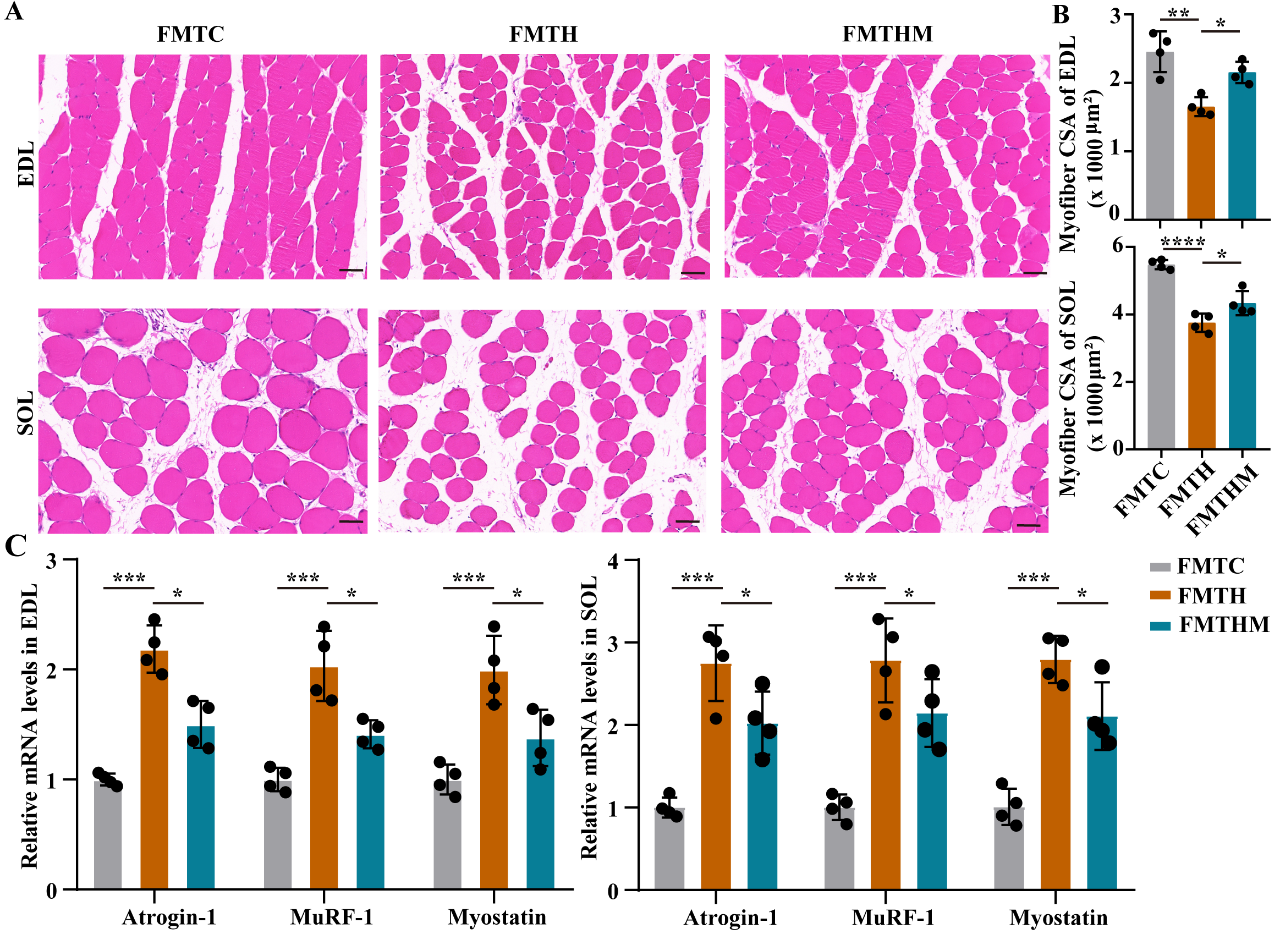


**Figure S12 Fecal suspension of MLT-treated rats inhibits muscle atrophy in recipient rats.** (A) The representative images of Hematoxylin and eosin (H&E) staining of EDL and SOL muscles (*n* = 4 rats/group), scale bar = 50 μm. (B) The cross-sectional area (CSA) of EDL and SOL muscles (*n* = 4 rats/group). (C) The mRNA expression levels of Atrogin-1, MuRF-1, and Myostatin in EDL and SOL muscles (*n* = 4 rats/group, repeated three times). Data were reported as mean ± s.e.m and analyzed by one-way ANOVA, followed by Tukey’s multiple comparisons test (B, C). **P*<0.05, ***P*<0.01, ****P*<0.001, *****P*<0.0001 versus the recipient rats received fecal suspension of HFD-fed rats.


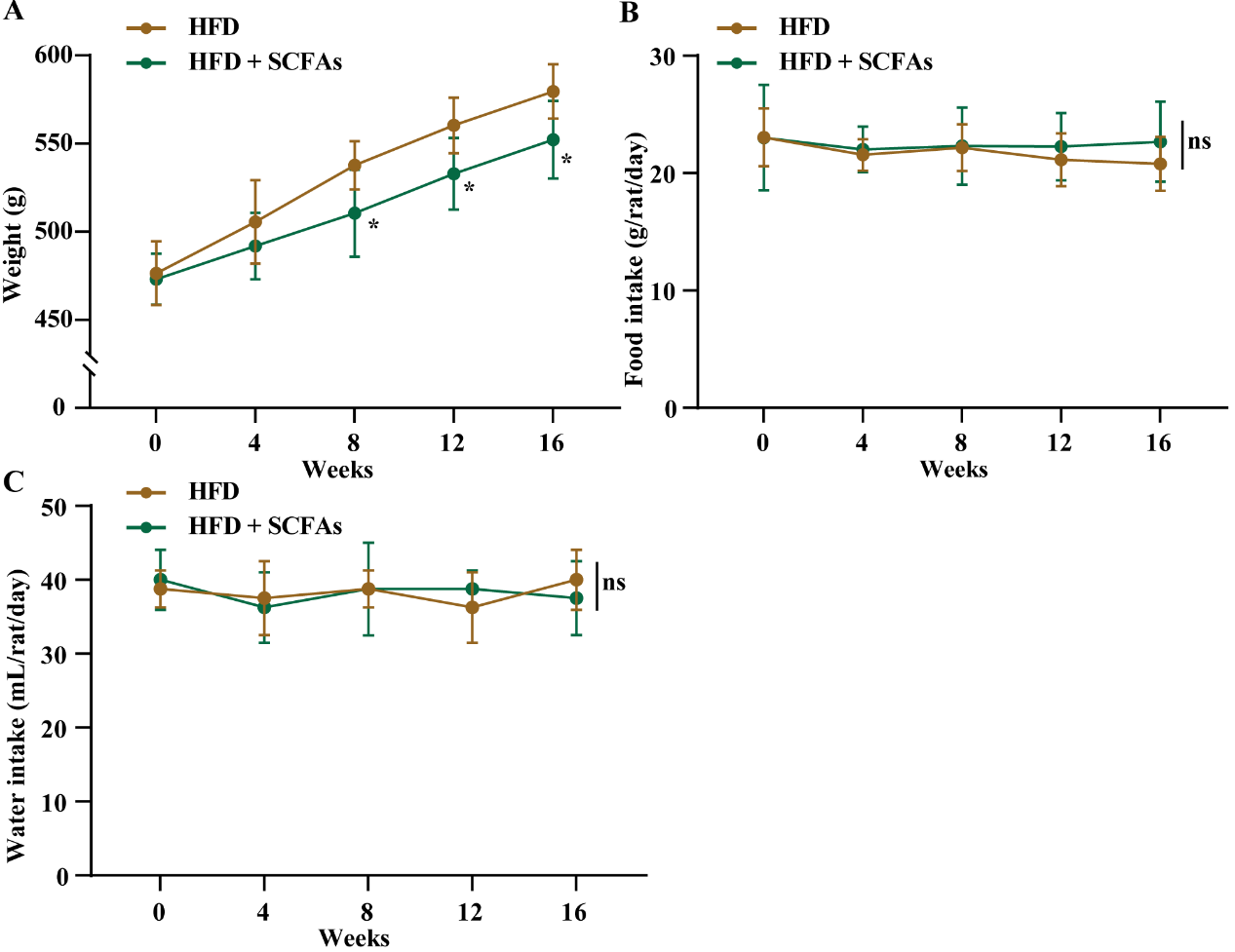


**Figure S13 SCFAs treatment ameliorates obesity in HFD-fed rats.** (A) Body weight (*n* = 8 rats/group). (B) Food intake (*n* = 8 rats/group). (C) Water intake (*n* = 8 rats/group). Data were analyzed by T-test (A–C). **P*<0.05 versus HFD-fed rats. ns: not significant.


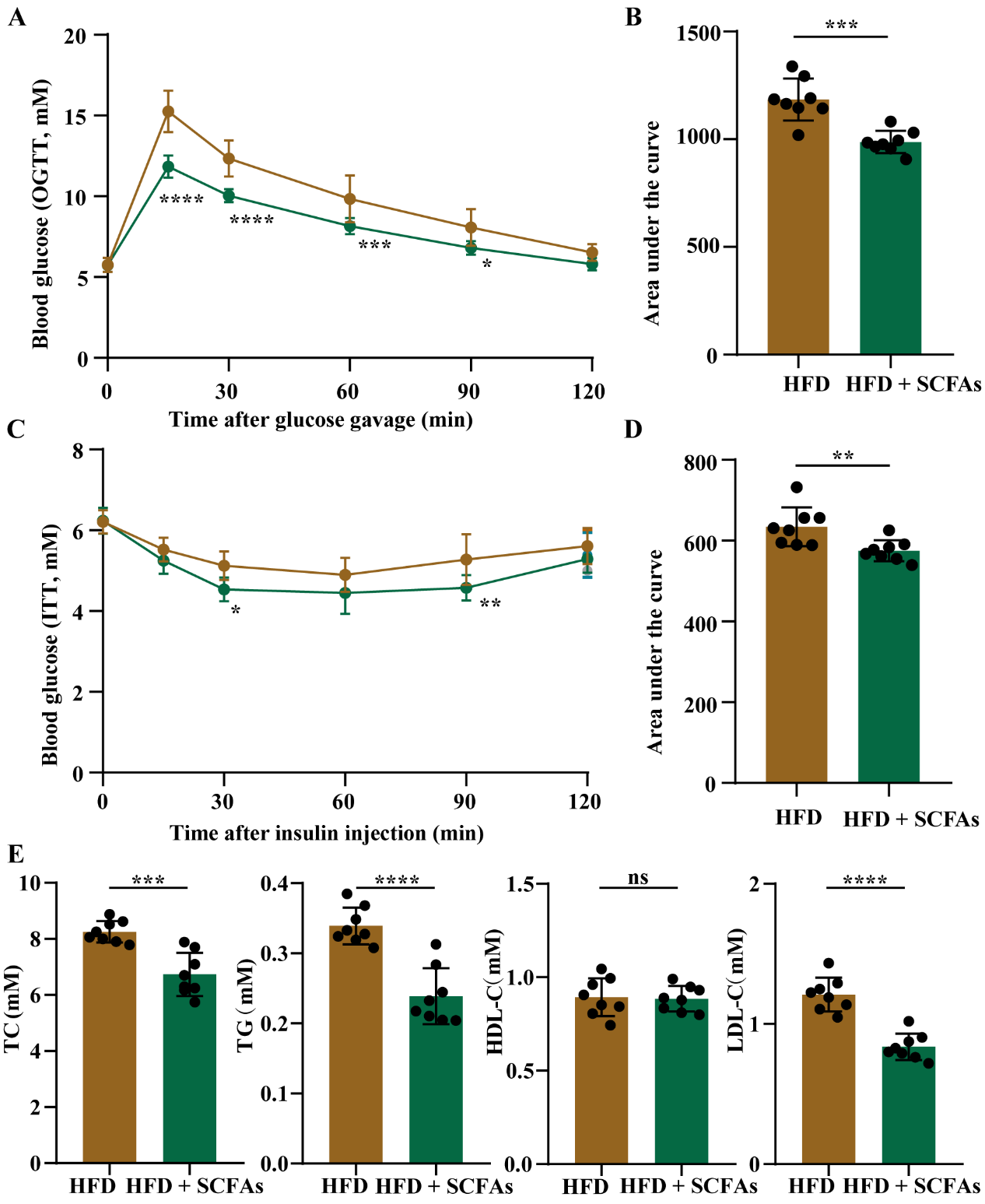


**Figure S14 SCFAs treatment alleviates glucose and lipid metabolism disorders in HFD-fed rats.** (A) The blood glucose at 0, 15, 30, 60, 90, and 120 mins after oral administrated with D-glucose solution (*n* = 8 rats/group). (B) The AUC value of blood glucose in OGTT (*n* = 8 rats/group). (C) The blood glucose at 0, 15, 30, 60, 90, and 120 mins after intraperitoneal injection with insulin (*n* = 8 rats/group). (D) The AUC value of blood glucose in iPITT (*n* = 8 rats/group). (E) The levels of TC, TG, HDL-C, and LDL-C in serum (*n* = 8 rats/group, repeated three times). Data were reported as mean ± s.e.m and analyzed by T-test (A–E). **P*<0.05, ***P*<0.01, ****P*<0.001, *****P*<0.0001 versus HFD-fed rats. ns: not significant.


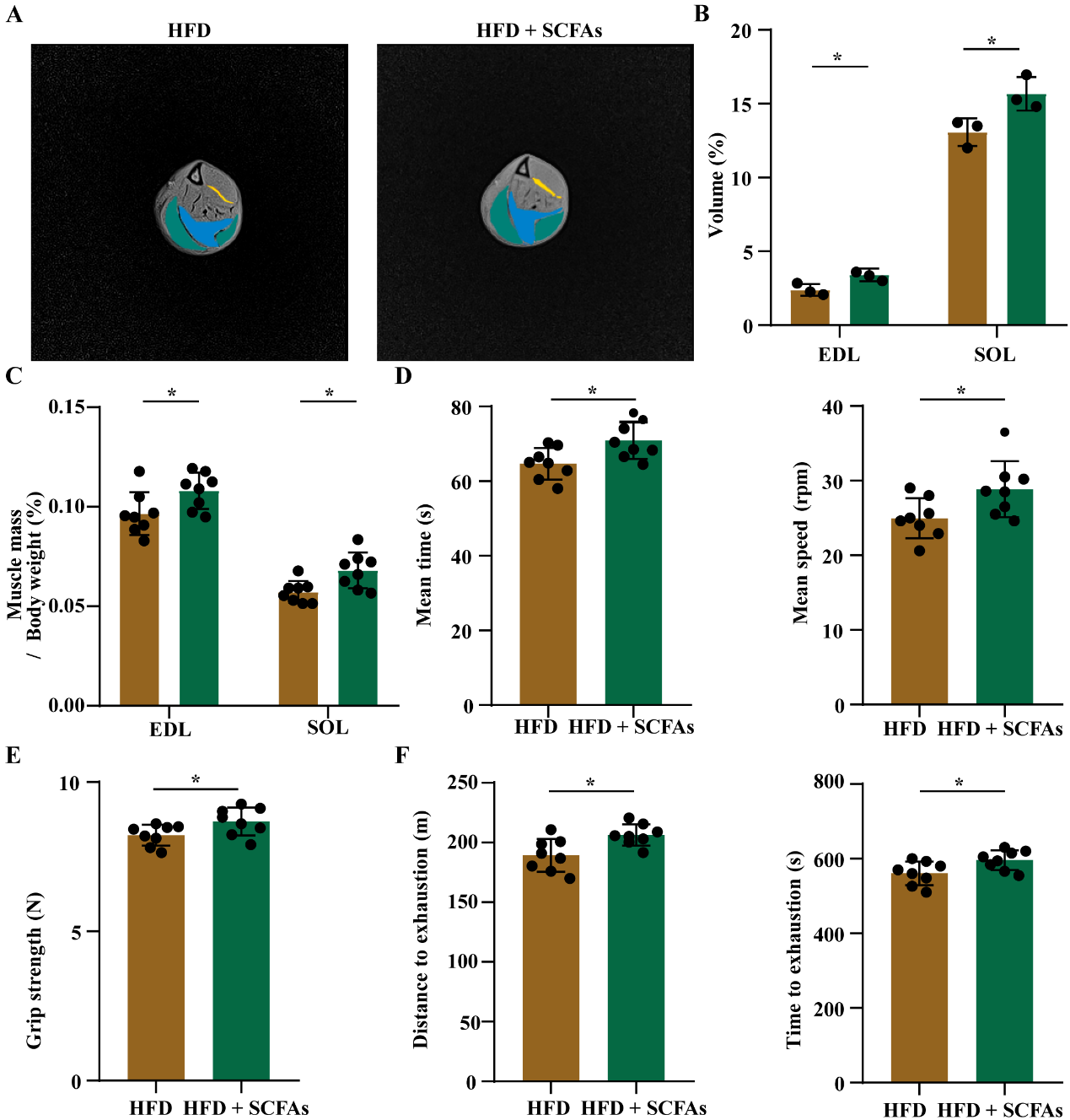


**Figure S15 SCFAs treatment improves muscle mass, strength, and function in HFD-fed rats.** (A) The representative images of gastrocnemius, EDL, and SOL muscles examined by MRI (*n* = 3 rats/group). Green represents gastrocnemius, blue represents SOL, and yellow represents EDL. (B) Quantitative analysis of muscle volume (*n* = 3 rats/group). (C) The weights of EDL and SOL muscles (*n* = 8 rats/group). (D) The mean time and speed of rats in the rotarod test (*n* = 8 rats/group). (E) Grip strength (*n* = 8 rats/group). (F) The total distance and total time of rats in the exhaustive running test (*n* = 8 rats/group). Data were reported as mean ± s.e.m and analyzed by T-test (A–F). **P*<0.05 versus HFD-fed rats.

**
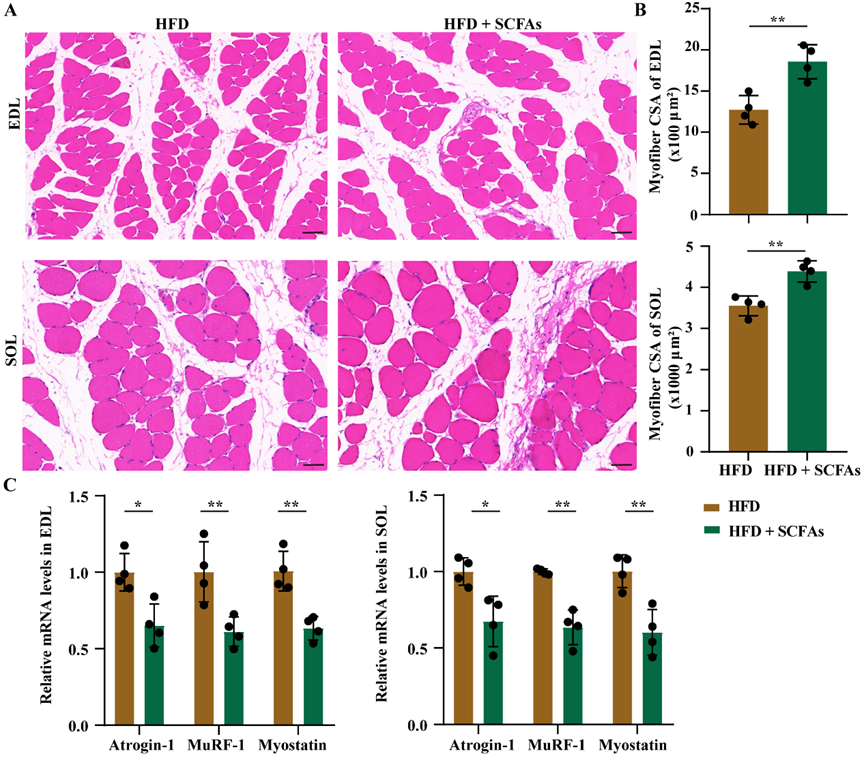
**

**Figure S1****6 SCFAs treatment mitigates muscle atrophy in HFD-fed rats.** (A) The representative images of H&E staining of EDL and SOL muscles (*n* = 4 rats/group), scale bar = 50 μm. (B) The CSA of EDL and SOL muscles (*n* = 4 rats/group). (C) The mRNA expression levels of Atrogin-1, MuRF-1, and Myostatin in EDL and SOL muscles (*n* = 4 rats/group, repeated three times). Data were reported as mean ± s.e.m and analyzed by T-test (B, C). **P*<0.05, ***P*<0.01 versus HFD-fed rats.
