# Supplementary material for: Melatonin Mitigates Sarcopenic Obesity via Microbiota and Short‐Chain Fatty Acids: Evidence From Epidemiologic and In Vivo Studies
Source: J Cachexia Sarcopenia Muscle. 2025 Jun 13;16(3):e13869. doi: 10.1002/jcsm.13869 (PMC12163512; doi:10.1002/jcsm.13869)
Supplement: Supplementary file 3 — Data S2 Supplementary Information. [file JCSM-16-e13869-s003.docx]

**Melatonin mitigates sarcopenic obesity via microbiota** **and short-chain fatty acids: Evidence from epidemiologic and *in vivo* studies**

**Journal of Cachexia Sarcopenia and Muscle**

Xiaoxing Mo^1^, Lihui Shen^1^, Xinyu Wang^1^, Wenqing Ni^2^, Linyan Li^1^, Lili Xia^1^, Hongjie Liu^1^, Ruijie Cheng^1^, Lin Wen^1^, Jian Xu ^2*^& Liegang Liu^1*^

^1^ Department of Nutrition and Food Hygiene, Hubei Key Laboratory of Food Nutrition and Safety, MOE Key Lab of Environment and Health, School of Public Health, Tongji Medical College, Huazhong University of Science and Technology, 13 Hangkong Road, Wuhan, 430030, China. [2024520214@hust.edu.cn](mailto:d202081565@hust.edu.cn), [M202275507@hust.edu.cn](mailto:M202275507@hust.edu.cn), [xywang_@hust.edu.cn,](mailto:xywang_@hust.edu.cn,) [d202181656@hust.edu.cn](mailto:d202181656@hust.edu.cn), [d202181612@hust.edu.cn,](mailto:d202181612@hust.edu.cn,) D201981405@hust.edu.cn, [d202381824@hust.edu.cn](mailto:d202381824@hust.edu.cn), [wenlin@hust.edu.cn](mailto:wenlin@hust.edu.cn), lgliu@mails.tjmu.edu.cn.

^2^ Department of Elderly Health Management, Shenzhen Center for Chronic Disease Control, Shenzhen, Guangdong, China. [wenqni@163.com](mailto:wenqni@163.com), anniexu73@126.com.

***Correspondence:**

Dr. Liegang Liu, Email: [lgliu@mails.tjmu.edu.cn](mailto:lgliu@mails.tjmu.edu.cn), Tel: +86 27 83650522, Fax: +86 27 83650522; Dr. Jian Xu, Email: [anniexu73@126.com](mailto:anniexu73@126.com).

**Supplementary references**

S1. Ni W, Peng X, Yuan X, Sun Y, Zhang H, Zhang Y, et al. Protocol for Shenzhen Ageing Cohort Study (SZ-ageing): a prospective observational cohort study of elderly disability and cognitive impairment. BMJ Open. 2023;13(1):e065761.

S2. Gan D, Wang X, Xu X, Kang Q, Lu Z, Jia M, et al. Sarcopenia and sarcopenic obesity after cholecystectomy: A population-based study. Obesity (Silver Spring). 2022;30(2):482-490.

S3. Petroni ML, Caletti MT, Dalle Grave R, Bazzocchi A, Aparisi Gomez MP, Marchesini G. Prevention and Treatment of Sarcopenic Obesity in Women. Nutrients. 2019;11(6).

S4. Fernandez-Martinez J, Ramirez-Casas Y, Aranda-Martinez P, Lopez-Rodriguez A, Sayed RKA, Escames G, et al. iMS-Bmal1(-/-) mice show evident signs of sarcopenia that are counteracted by exercise and melatonin therapies. J Pineal Res. 2024;76(1):e12912.

S5. Jimenez-Aranda A, Fernandez-Vazquez G, Mohammad ASM, Reiter RJ, Agil A. Melatonin improves mitochondrial function in inguinal white adipose tissue of Zucker diabetic fatty rats. J Pineal Res. 2014;57(1):103-109.

S6. Favero G, Golic I, Arnaboldi F, Cappella A, Korac A, Monsalve M, et al. Cardiometabolic Changes in Sirtuin1-Heterozygous Mice on High-Fat Diet and Melatonin Supplementation. Int J Mol Sci. 2024;25(2).

S7. Li A, Nelson SR, Rahmanseresht S, Braet F, Cornachione AS, Previs SB, et al. Skeletal MyBP-C isoforms tune the molecular contractility of divergent skeletal muscle systems. Proc Natl Acad Sci U S A. 2019;116(43):21882-21892.

S8. Mo X, Shen L, Cheng R, Wang P, Wen L, Sun Y, et al. Faecal microbiota transplantation from young rats attenuates age-related sarcopenia revealed by multiomics analysis. J Cachexia Sarcopenia Muscle. 2023;14(5):2168-2183.

S9. Lahiri S, Kim H, Garcia-Perez I, Reza MM, Martin KA, Kundu P, et al. The gut microbiota influences skeletal muscle mass and function in mice. Sci Transl Med. 2019;11(502).

S10. Zhang Z, Hu Y, Zhang N, Li J, Lu J, Wei H. Dietary supplementation with non-digestible isomaltooligosaccharide and Lactiplantibacillus plantarum ZDY2013 ameliorates DSS-induced colitis via modulating intestinal barrier integrity and the gut microbiota. Food Funct. 2024;15(11):5908-5920.

S11. Hays KE, Pfaffinger JM, Ryznar R. The interplay between gut microbiota, short-chain fatty acids, and implications for host health and disease. Gut Microbes. 2024;16(1):2393270.

S12. Zhou R, Liu T, Qin Y, Xie J, Zhang S, Xie Y, et al. Polygonatum cyrtonema Hua polysaccharides alleviate muscle atrophy and fat lipolysis by regulating the gut microenvironment in chemotherapy-induced cachexia. Front Pharmacol. 2025;16:1503785.

S13. Zou XY, Zhang M, Tu WJ, Zhang Q, Jin ML, Fang RD, et al. Bacillus subtilis inhibits intestinal inflammation and oxidative stress by regulating gut flora and related metabolites in laying hens. Animal. 2022;16(3):100474.

S14. Paone P,Cani PD. Mucus barrier, mucins and gut microbiota: the expected slimy partners? Gut. 2020;69(12):2232-2243.

S15. Martin-Gallausiaux C, Marinelli L, Blottiere HM, Larraufie P, Lapaque N. SCFA: mechanisms and functional importance in the gut. Proc Nutr Soc. 2021;80(1):37-49.

S16. Van Hul M, Neyrinck AM, Everard A, Abot A, Bindels LB, Delzenne NM, et al. Role of the intestinal microbiota in contributing to weight disorders and associated comorbidities. Clin Microbiol Rev. 2024:e0004523.
