# Supplementary material for: Melatonin Mitigates Sarcopenic Obesity via Microbiota and Short‐Chain Fatty Acids: Evidence From Epidemiologic and In Vivo Studies
Source: J Cachexia Sarcopenia Muscle. 2025 Jun 13;16(3):e13869. doi: 10.1002/jcsm.13869 (PMC12163512; doi:10.1002/jcsm.13869)
Supplement: Supplementary file 4 — Table S1 The primer sequences of muscle atrophy‐related genes. Table S2. Clinical characteristics of participants in this study. [file JCSM-16-e13869-s004.docx]

**Melatonin mitigates sarcopenic obesity via microbiota** **and short-chain fatty acids: Evidence from epidemiologic and *in vivo* studies**

**Journal of Cachexia Sarcopenia and Muscle**

Xiaoxing Mo^1^, Lihui Shen^1^, Xinyu Wang^1^, Wenqing Ni^2^, Linyan Li^1^, Lili Xia^1^, Hongjie Liu^1^, Ruijie Cheng^1^, Lin Wen^1^, Jian Xu ^2*^& Liegang Liu^1*^

^1^ Department of Nutrition and Food Hygiene, Hubei Key Laboratory of Food Nutrition and Safety, MOE Key Lab of Environment and Health, School of Public Health, Tongji Medical College, Huazhong University of Science and Technology, 13 Hangkong Road, Wuhan, 430030, China. [2024520214@hust.edu.cn](mailto:d202081565@hust.edu.cn), [M202275507@hust.edu.cn](mailto:M202275507@hust.edu.cn), [xywang_@hust.edu.cn,](mailto:xywang_@hust.edu.cn,) [d202181656@hust.edu.cn](mailto:d202181656@hust.edu.cn), [d202181612@hust.edu.cn,](mailto:d202181612@hust.edu.cn,) D201981405@hust.edu.cn, [d202381824@hust.edu.cn](mailto:d202381824@hust.edu.cn), [wenlin@hust.edu.cn](mailto:wenlin@hust.edu.cn), lgliu@mails.tjmu.edu.cn.

^2^ Department of Elderly Health Management, Shenzhen Center for Chronic Disease Control, Shenzhen, Guangdong, China. [wenqni@163.com](mailto:wenqni@163.com), anniexu73@126.com.

***Correspondence:**

Dr. Liegang Liu, Email: [lgliu@mails.tjmu.edu.cn](mailto:lgliu@mails.tjmu.edu.cn), Tel: +86 27 83650522, Fax: +86 27 83650522; Dr. Jian Xu, Email: [anniexu73@126.com](mailto:anniexu73@126.com).

**Supplementary tables**

Table S1. The primer sequences of muscle atrophy-related genes

| Gene | Forward | Reverse |
| --- | --- | --- |
| MuRF-1 | TGACCAAGGAAAACAGCCACCAG | TCACTCCTTCTTCTCGTCCAGGATGG |
| Atrogin | TACTAAGGAGCGCCATGGATACT | GTTGAATCTTCTGGAATCCAGGAT |
| Myostatin | CTACCACGGAAACAATCATTACCA | AGCAACATTTGGGCTTTCCAT |
| GAPDH | GGATCTCGCTCCTGGAAGATG | TACCAGGGCTGCCTTCTCTTG |

Table S2. Clinical characteristics of participants in this study

| Variable | Control  (n = 31) | SO  (n = 31) | P-value |
| --- | --- | --- | --- |
| Age, years | 70.26 ± 4.19 | 70.58 ± 4.34 | 0.750 |
| Male | 15 (48.38%) | 14 (45.16%) | 0.803 |
| BMI, kg/m^2^ | 23.81 ± 3.44 | 24.28 ± 2.37 | 0.530 |
| BF, % | 28.78 ± 7.84 | 36.22 ± 5.81 | < 0.001 |
| ASMI | 6.79 ± 0.95 | 5.88 ± 0.80 | < 0.001 |
| Handgrip strength | 27.94 ± 9.06 | 21.97 ± 9.39 | 0.013 |
| SPPB score | 11.00 (10.00, 12.00) | 9.00 (8.00, 11.00) | < 0.001 |

Data are shown as mean ± SD, median (quartile) or number (percentage). SO, sarcopenic obesity; BMI, body mass index; BF, body fat; ASMI, appendicular skeletal muscle mass index; SPPB, short physical performance battery.
